# Circ0083429 Regulates Osteoarthritis Progression via the Mir-346/*SMAD3* Axis

**DOI:** 10.3389/fcell.2020.579945

**Published:** 2021-01-15

**Authors:** Teng Yao, Yute Yang, Ziang Xie, Yining Xu, Yizhen Huang, Jun Gao, Shuying Shen, Huali Ye, Yasaman Iranmanesh, Shunwu Fan, Jianjun Ma

**Affiliations:** ^1^Department of Orthopaedic Surgery, Sir Run Run Shaw Hospital, Zhejiang University School of Medicine, Hangzhou, China; ^2^Key Laboratory of Musculoskeletal System Degeneration and Regeneration Translational Research of Zhejiang Province, Hangzhou, China; ^3^School of Medicine, Shaoxing University, Shaoxing, China

**Keywords:** chondrocytes, Circ0083429, miR-346, osteoarthritis, *SMAD3*

## Abstract

Osteoarthritis (OA) is a degenerative joint disease. Currently, apart from symptomatic treatment or joint replacement, no other effective treatments for OA exist. The mechanisms underlying OA remain elusive and require further research. Circular RNAs (circRNAs) are known to be involved in many diseases; however, their function in OA is not yet fully understood. Here, we identified a novel circRNA, Circ0083429. The role of Circ0083429 in OA was confirmed via western blot (WB), quantitative real-time PCR (qRT-PCR), and immunofluorescence (IF) through knockdown and overexpression experiments. The binding of Circ0083429 to downstream miR-346 and its target gene SMAD3 was predicted via bioinformatics analysis and verified using a luciferase reporter assay and RNA pulldown experiments. Finally, the function of Circ0083429 was evaluated in mouse OA models. In our study, we found that Circ0083429 regulates the homeostasis of the extracellular matrix (ECM) in human chondrocytes. Mechanistically, Circ0083429 affects OA by regulating the mRNA level of *SMAD3* through the sponging of microRNA (miRNA)-346. Injecting adeno-associated virus Circ0083429 into the intra-junction of the mouse knee alleviated OA. In conclusion, Circ0083429 regulates the ECM via the regulation of the downstream miRNA-346/*SMAD3* in human chondrocytes, which provides a new therapeutic strategy for OA.

## Introduction

Osteoarthritis (OA) is a degenerative disease associated with abnormal changes in the joint cartilage. OA negatively affects a patient’s quality of life, especially among elderly individuals (Hayami et al., 2006; Jiang and Tuan, 2015). It is one of the leading causes of disability, socioeconomic burden, and chronic pain worldwide (Cross et al., 2014; Glyn-Jones et al., 2015). Therefore, it is necessary to explore the mechanisms underlying OA pathogenesis and progression. It has been shown that an imbalance in the homeostasis of the extracellular matrix (ECM) is associated with OA (Mobasheri et al., 2017); however, the underlying regulatory mechanisms have yet to be elucidated (Goldring, 2000b, 2006; Lotz, 2001; Sinusas, 2012).

In recent years, the functions of circular RNAs (circRNAs) in various diseases have garnered increasing attention, and circRNAs have been suggested as markers for disease diagnosis (Lu and Xu, 2016; Ryu et al., 2020). CircRNAs are non-coding RNA molecules that do not possess a 5′-end cap and a 3′-end poly(A) tail, as they are covalently bonded to form a circular structure (Di Timoteo et al., 2020). The circular structure is not sensitive to RNase R and is more stable than linear RNA. In addition, circRNAs are evolutionarily conserved, and their expression shows tissue and spatio-temporal specificity (Veno et al., 2015). CircRNAs have functions mainly through the following three mechanisms: (1) as microRNA (miRNA) sponges, the circRNAs competitively bind to miRNAs to regulate downstream gene expression; (2) circRNAs bind with RNA binding proteins to regulate the transcription of the linear parent genes or to regulate protein functions as well as RNA translation into peptides (Qu et al., 2015; Hsiao et al., 2017; Meng et al., 2017); and (3) CircRNAs are also able to combine with ribosomes to affect translation, which in turn regulates cellular functions (Salzman, 2016; Han et al., 2017; Pamudurti et al., 2017; Yu et al., 2018). CircRNAs have been shown to play a regulatory role in tumor cells, but there are only a few reports on their function in chondrocytes. An in-depth study of the function and mechanism of non-coding RNAs in chondrocytes is very important for evaluating their use as a key target for the prevention and treatment of OA.

Recent studies have demonstrated that patients with or without OA exhibit different circRNA expression patterns (Shen et al., 2019). This suggests a potential function of circRNAs in OA. We performed a literature review to identify the differential expression patterns of circRNAs in OA and non-OA tissue (Li et al., 2019). Based on this literature review, we found that *hsa_circ_0083429* (Circ0083429), oriented from *8:17543318| 17543715* of chromosome 8, was evidently downregulated in OA tissue (Shen et al., 2019). A previous study showed that circRNA.33186 contributes to the pathogenesis of OA by sponging miR-127-5p (Zhou et al., 2019). This suggests that circRNAs may act as small molecules that participate in the progression of OA and could be potential targets for OA treatment.

MiRNAs are a class of single-stranded non-coding RNAs that are approximately 22 nucleotides in length and are encoded by endogenous genes. Due to their role in post-transcriptional gene regulation in both animals and plants (Lu and Rothenberg, 2018), they are involved in the intrinsic molecular regulation of various diseases, including OA, by regulating various cellular and molecular functions (Hu et al., 2018; Malemud, 2018).

In this study, we identified Circ0083429, a novel circRNA, which regulated the progression of OA by sponging miR-346. Thus, this circRNA may act as a potential target for the treatment of OA.

## Materials and Methods

### Ethical Consideration

Animal experiments were conducted following the guidelines of the National Institutes of Health. The Ethics Committee of Sir Run Run Shaw Hospital of Zhejiang University, China, approved all animal experiments. All animal experiments were in accordance with the guidelines for the care, treatment, and euthanasia of experimental animals. This study complied with the standards of the Ethics Committee on Human Experimentation of the Sir Run Run Shaw Hospital of Zhejiang University. Informed consent was obtained from the patients involved in this study.

### Collection of Human Cartilage and Cell Culture

Human chondrocytes were derived from 20 patients who underwent joint replacements for OA. These patients had no history of any other underlying diseases. The intercondylar cartilage consists of a non-weight-bearing area, as control cartilage, and a weight-bearing area, which is considered osteoarthritic cartilage. Cartilage cells from all patients were taken from the same tissue site and used separately. The position of the articular cartilage and patient information are shown in Supplementary Figures 1A,B. The cartilage tissue was shredded using disinfected instruments and incubated with type 2 collagenase (Sigma, CA, United States) for 6 h in a 37°C cell incubator. The residue was removed by filtration, followed by centrifugation at 1,000 rpm for 5 min to remove the supernatant. The cell pellet was resuspended and washed three times with phosphate-buffered saline (PBS). Cells were then transferred into a 10-cm petri dish and cultured in Dulbecco’s modified Eagle’s medium (DMEM) with 10% fetal bovine serum (FBS; Gibco, Grand Island, NY, United States). All cells were used within three generations. HEK-293T cells [American Type Culture Collection (ATCC): CRL-1573), and SW1353 cells were obtained from the ATCC (Manassas, VA, United States) and cultured in DMEM with 10% FBS. All cells were grown in a 37°C incubator with 5% CO_2_.

### Small Interfering RNAs and Stable Transfection

Small Interfering RNAs (siRNAs) were designed by RiboBio (Guangzhou, China). SiRNAs were mixed with Lipofectamine iMAX (Invitrogen, CA, United States) for 5 min in Opti-MEM (Gibco) and then transferred into the medium containing chondrocytes at 50% confluency. Total RNA and protein were extracted and used in the subsequent experiments. The PLKO.1 plasmid was used to construct the short hairpin RNA (shRNA)–Circ0083429 plasmids. The structure of PLKO.1 is shown in Supplementary Figure 3A. We used the target plasmid, packaging plasmid, and membrane plasmid to co-transfect the 293T cells. After 48 h, the supernatant virus solution of the 293T medium was collected. After filtration, the chondrocytes were infected with the virus solution and inspected with a fluorescence microscope. Puromycin was used to select resistant cells. RNA was extracted to verify the efficiency of the transfection. Stably expressed chondrocytes were used within three generations.

### Construction of the Overexpression Cell Line

Lentiviral overexpression plasmids were purchased from Hanbio (Wuhan, China). The structure of the plasmids is shown in Supplementary Figures 3B,C. We used the target plasmid, packaging plasmid, and membrane plasmid to co-transfect the HEK-293T cells. After 48 h, the supernatant virus solution of the transfected HEK-293T was collected and moved on the adherent primary chondrocytes after filtration to remove the cell debris. After another 72 h, the positive transfected chondrocytes were observed with green fluorescence under a fluorescence microscope and selected using puromycin (100 μg/ml). Then, total RNA was extracted to verify the efficiency of overexpression. The control chondrocytes were transfected with the lentiviral solution formed by HEK-293T cells transfected with blank vector plasmid along with packaging plasmid and membrane plasmid. All overexpressed chondrocytes were used only within three generations.

### Interleukin-1β Treatment

Interleukin (IL)-1β was purchased from R&D Systems (Minnesota America). IL-1β stimulated cells at a final concentration of 10 ng/ml. Total RNA was extracted after IL-1β treatment for 24 h. Protein was extracted after IL-1β treatment for 48 h.

### Cell Counting Kit-8 Assay

Human chondrocytes were seeded into a 96-well plate (90 μl/well) at a density of 1 × 10^5^ cells/ml in an incubator with 37°C and 5% CO_2_ for pre-culture. After treatment for 24, 48, 72, and 96 h, 10 μl of Cell Counting Kit-8 (CCK-8) solution was added to each well, and the plate was incubated for 2 h. The absorbance was then measured at 450 nm using a microplate reader (Spectrophotometer, Thermo Fisher, MA, United States).

### Fluorescence *in situ* Hybridization

Alexa Fluor 555-labeled Circ0083429 and Alexa Fluor 488-labeled miR-346 probes were purchased from Guangzhou RiboBio. Probe signals were measured using a fluorescence *in situ* hybridization (FISH) kit (RiboBio). Images were acquired using a fluorescence microscope (Nikon SMZ18, Japan). The sequence of the Circ0083429 probe was 5′-CY3-GTCTGACG AAGGCGACCTATCACATACGATAAA-3′. The sequence of the miR-346 probe was 5′-FAM-AGAGGCAGGCATGCGGGC AGACA-FAM-3′.

### RNA Immunoprecipitation

RNA immunoprecipitation (RIP) experiments were performed using a RIP Kit (Millipore, Bedford, MA, United States). Chondrocytes were subjected to argonaute RISC catalytic component 2 (AGO-2)-RIP. Chondrocytes (1 × 10^6^ cells) were centrifuged and resuspended in RIP lysis buffer (100 μl) with RNase inhibitors and a protease inhibitor cocktail. The lysates were then incubated with Igg or anti-Ago2 antibody beads. The mixture of lysates and magnetic beads was rotated overnight at 4°C. The lysates were treated with proteinase K buffer, and the immunoprecipitated RNA was extracted using an RNeasy MinElute Cleanup Kit (Qiagen, Dusseldorf, Germany). The purified RNA was used to detect Circ0083429 levels via reverse transcription (TaKaRa, Tokyo, Japan) and quantitative real-time polymerase chain reaction (qRT-PCR). CircSERPINE2 (Shen et al., 2019) acted as a positive control.

### Luciferase Reporter Assay

PGL3-basic plasmids containing wild-type (WT) or mutant (Mut) Circ0083429 were constructed by the GeneChem company (Shanghai, China). Renilla luciferase (RU2) was inserted upstream of the target gene, while firefly luciferase (RU1) was inserted downstream. The recombinant plasmids and miR-346 mimic were transfected into HEK-293T cells. The experimental groups were as follows: control vector + negative control (NC) mimic, control vector + miR-346 mimic, WT or Mut plasmid + NC mimic, and WT or Mut plasmid + miR-346 mimic. A luminometer (Thermo Fisher, MA, United States) was used to detect the fluorescent activity. RU1 activity and RU2 activity were measured to calculate the RU1/RU2 ratio. Fold change refers to the multiple relationships of RU1/RU2 ratio between each group and the control vector + NC mimic group.

PGL3-basic plasmids containing WT or Mut *SMAD3* were constructed by Shanghai GeneChem. RU2 was inserted upstream of the target gene, while RU1 was inserted downstream. The recombinant plasmids and miR-346 mimic were transfected into HEK-293T cells. The experimental groups were as follows: WT or Mut plasmid + NC mimic, and WT or Mut plasmid + miR-346 mimic. A luminometer (Tuner BioSystems’ instrument) was used to detect the fluorescent activity. RU1 activity and RU2 activity were measured to calculate the RU1/RU2 ratio. Fold change refers to the multiple relationships of RU1/RU2 ratio between each group and the Mut plasmid + NC mimic group.

### Prediction of MicroRNA Targets of Circ0083429

MiRNAs capable of binding Circ0083429 were predicted using miRanda^1^, RNAhybrid^2^, and TargetScan^3^. The following filtering restrictions were used: (i) total energy < 20 kcal/mol and total score ≥ 140; (ii) minimum free energy (MFE) ≤ 25 kcal/mol; and (iii) number of estimated binding sites ≥ 1. We considered the intersection of the three database predictions.

### RNA Pulldown Assay

The Circ0083429 probe was designed by RiboBio. Human chondrocytes (1 × 10^6^) transfected with the Circ0083429 overexpression construct were harvested and lysed with lysis buffer [50 mM of Tris-HCl pH 7.0, 10 mM of ethylenediaminetetraacetic acid (EDTA), 1% sodium dodecyl sulfate (SDS) supplemented with 200 U/ml of a RNAse inhibitor solution, and a cocktail of proteases inhibitor 5 μl/ml]. A mixture of Circ0083429 probe or oligo probe and C-1 magnetic beads (Life Technologies) was incubated at 25°C for 2 h. Then, the probe-coated beads were incubated with the cell lysates at 4°C overnight. Finally, the RNA bound to the beads was washed with wash buffer [SDS 0.5%, saline sodium citrate (SSC) 2×], and RNA was eluted with elution buffer (10 mM of Tris-HCl pH 7.0, 100 mM of NaCl, 1 mM of EDTA, 0.5% SDS, and 5 μl of 20 mg/ml proteinase-K). And the extracted RNA was used for qRT-PCR analysis. The Circ0083429 probe sequence was 5′-GTCTGACGAAGGCGAC CTATCACATACGATAAA-3′. The oligonucleotide probe sequence was 5′-CAGACTGCTTCCGCTGGATAGTGTATGC TATTT-3′.

### Prediction of MiR-346 Target Genes

MiR-346 binding sites were predicted using miRNA data integration portal (miRDIP)^4^. In total, 19 636 predicted bound target genes were found. After the score class was filtered, 382 genes were associated with miR-346 in the highest score class. Subsequently, the Kyoto Encyclopedia of Genes and Genomes (KEGG) pathway enrichment analysis was performed with these 382 genes, and the results suggested 29 signaling pathways related to these genes, and the most relevant was the TGF-β signaling pathway. In this pathway, eight genes were predicted to bind to miR-346. They are ROCK1, ACVR2B, ACVR1, SMAD3, LTBP1, MAPK1, BMP8B, and CDKN2B. We used mimic miR-346 to screen these eight genes. The KEGG analysis was performed based on the results obtained from the Database for Annotation, Visualization, and Integrated Discovery (DAVID)^5^.

### Immunofluorescence

Cells were permeabilized for 30 min in 0.5% Triton X-100 after being fixed in 4% paraformaldehyde for 30 min. Then, the cells were blocked in 5% bovine serum albumin (BSA) for 1 h. Primary antibodies were diluted 1:100 in 5% BSA and incubated with fixed cells at 4°C overnight. The cells were then washed five times with PBS and incubated for 1 h at room temperature (25°C) with CL594- or CL488-conjugated secondary antibodies diluted at 1:300 in PBS. Finally, the cells were washed five more times with PBS and protected from light. Immunofluorescent images were acquired using a Colibri epifluorescence microscope (Carl Zeiss, Jena, Germany).

### Immunohistochemical, Alcian Blue, and Safranin O Fast Staining

Cartilage tissue was obtained from patients with OA and mice and was fixed in 4% paraformaldehyde. For patient’s cartilage tissue, the samples were embedded in paraffin for 1 day and then sectioned. Each paraffin-embedded cartilage sample was sectioned at 4 μm. And the section was stained with 0.001% Fast Green solution and 0.1% safranin O solution (Sigma-Aldrich, St. Louis, MO, United States). For mice, the knee joint was embedded in paraffin after being stored in decalcifying solution for 1 month at 4°C. Paraffin blocks were sectioned using a microtome and placed on a slide. Sections were incubated with primary antibodies overnight at 4°C. After being twice washed with buffer, sections were incubated with secondary antibodies (Beyotime Institute of Biotechnology, Inc., Jiangsu, China) for 2 h at room temperature. Positively stained cells on knee joint surface were counted and calculated by Image-Pro Plus 6.0.

### Northern Blot

Formaldehyde agarose gels were used in electrophoresis for northern blot, and the RNA was transferred onto the membranes by 20 × SSPE (3.0 M of NaCl, 0.2 M of NaH_2_PO_4_, and 0.02 M of EDTA at pH 7.4). Following this, a blocking reagent was applied to block endogenous biotin. After hybridization with pre-hybridization solution, 50 μl of the probe was directly added to the hybridization solution, after which the membranes were washed first with washing solution 1 and then washing solution 2 for 30 min at 65°C each. Finally, the membranes were exposed to Bio-Rad’s ECL solution (Beyotime, Shanghai, China). The sequences of Circ0083429 and CircSERPINE2 were designed by Ginbio company (Guangzhou, China). The probes for Circ0083429 and CircSERPINE2 were labeled using T7 RNA Polymerase *in vitro*, which was designed by Ginbio company (Guangzhou, China). The sequence of Circ0083429 is TAA TACGACTCACTATAGGGCTGCTTATCCACCATGGACAGA AAGATGGTCTGGGATCGTC. The total RNA was treated with RNase R (5 U/μg) for degradation at 37°C for 15 min, and the sequence of CircSERPINE2 is TAATACGACTCAC TATAGGTCATCTGTGCAGACTGAGTTTGTAGATGGGGCA GAGGAATCT.

### Agarose Gel Electrophoresis

The agarose powder was dissolved with TAE (242 g of TRIS base, 57.1 ml of glacial acetic acid, and 100 ml of 0.05 mol/L EDTA), and 1% agarose gel was prepared. The amplified samples were added to the gel and electrophoresed at 120 V for 35 min. After being stained with ethidium bromide (EB), the gel was exposed by Bio-Rad ChemiDoc XRS + Imaging system (CA, United States).

### Western Blot

Cells were lysed in radioimmunoprecipitation assay (RIPA) lysis buffer (MA0151, MeilunBio, China) with a protease inhibitor cocktail (FD1001, FDbio, Hangzhou, China) for 20 min on ice. After 20 min, 5× loading buffer (FD002, FDbio) was added to the lysis buffer. Protein expression was detected by SDS–polyacrylamide gel electrophoresis (SDS-PAGE) at 80 V for 15 min and 120 V for 90 min. The proteins were then transferred from the gel onto a polyvinylidene difluoride (PVDF) membrane (Amersham Bioscience, NJ) with transfer buffer at 300 mA for 100 min. The PVDF membrane was blocked with Tris-buffered saline and Tween 20 (TBST) containing 5% dissolved skim milk powder at room temperature for 1 h. Next, the PVDF membrane was incubated with primary antibodies overnight at 4°C. Then, the membrane was rinsed three times with TBST for 10 min. A horseradish peroxidase (HRP)-conjugated secondary antibody (FDM007 and FDR007, FDbio) was then added to the membrane and incubated for 1 h at room temperature. Finally, protein levels were detected using an enhanced chemiluminescence kit (FD8030, FDbio). Abcam (Cambridge, United Kingdom) provided anti-β-actin, anti-MMP3, anti-MMP13, anti-COL2A1, anti-aggrecan, and anti-ADAMTS4 antibodies. Primary antibody dilution buffer was provided by MeilunBio (Dalian, China).

### RNA Extraction and Quantitative Real-Time PCR Analysis

Total RNA was extracted from 143B, HOS cells, or tissues. Cells or tissues were treated with TRIzol reagent (Invitrogen, Carlsbad, CA, United States) according to the manufacturer’s instructions. RNA was stored at −80°C. RNA was used to proceeding reverse transcription using Evo M-MLV RT Premix for qPCR Kit (Agbio, Hunan, China) according to manufacturer’s instruction. Amplification was performed with 10 μl reaction volume containing 5 μl of SYBR Green Premix (Agbio, Hunan, China), 1 μl of primer (Tsingke Bio, Hangzhou, China), 1 μl of cDNA, and RNase-free water. The amplification was detected by an ABI 7500 Sequencing Detection System (Applied Biosystems, Foster City, CA, United States). All experiments were carried out in three biological replicates, and the results were normalized to the ACTIN. The sequence of primers is in Supplementary Table 3.

### RNase R Treatment Assay

RNA was extracted from cells and tissues using TRIzol (Invitrogen, Carlsbad, United States), as per the manufacturer’s instructions for the universal column RNA extraction kit (CWbio, Jiangsu, China). The RNA was divided into two groups: 2 mg of RNA was incubated at 37°C for 15 min with RNase R (Epicenter Technologies, Madison, WI, United States), whereas another 2 mg of RNA was incubated at 37°C for 15 min without RNase R. The expression of circRNAs and mRNAs was then detected by qRT-PCR, with β-actin serving as a control. For the miRNA assay, the samples were synthesized using the Mir-X miR First-Strand Synthesis Kit (TaKaRa) after removing the DNA with DNase I. cDNA expression was detected by qRT-PCR. The internal standard control was U6.

### Osteoarthritis Model and Injection of Circ0083429

We used 24 six-week-old C57BL mice. The mice were divided into four groups, each with six mice. At 6 weeks of age, mice from three groups underwent an anterior cruciate ligament resection surgery on both knee joints for the establishment of OA models, whereas one group of mice, the control group, did not undergo the surgery. After 4 weeks, two of the surgery groups were selected. Adeno-associated virus (AAV) Circ0083429 overexpression (WT) and AAV Circ0083429 mutant overexpression (MUT) were injected into the joint cavities of the modeled mice. In these two groups, when the mice were 11 weeks old, the AAV was injected again into the joint cavities of the mice. When the mice were 12 weeks old, they were sacrificed. The left knee joints of all the mice were collected for RNA and protein extraction, and the right knee joints of the mice were collected for immunohistochemistry. The structure of the adenovirus expression plasmid is shown in Supplementary Figure 3D.

### Statistical Analysis

All western blot and q-PCR experiments were repeated more than three times, while the other experiments were repeated at least three times. The most representative results are shown in the figures. The datasets of the different groups were analyzed by Student’s *t*-test or two-way analysis of variance. *P*-values ≤ 0.05 were considered to be statistically significant differences and are marked as ^∗^ in the figures. GraphPad Prism (version 7.0) was used to analyze the data.

## Results

### Patients With Osteoarthritis Exhibit Lower Circ0083429 Expression

**FIGURE 1:**
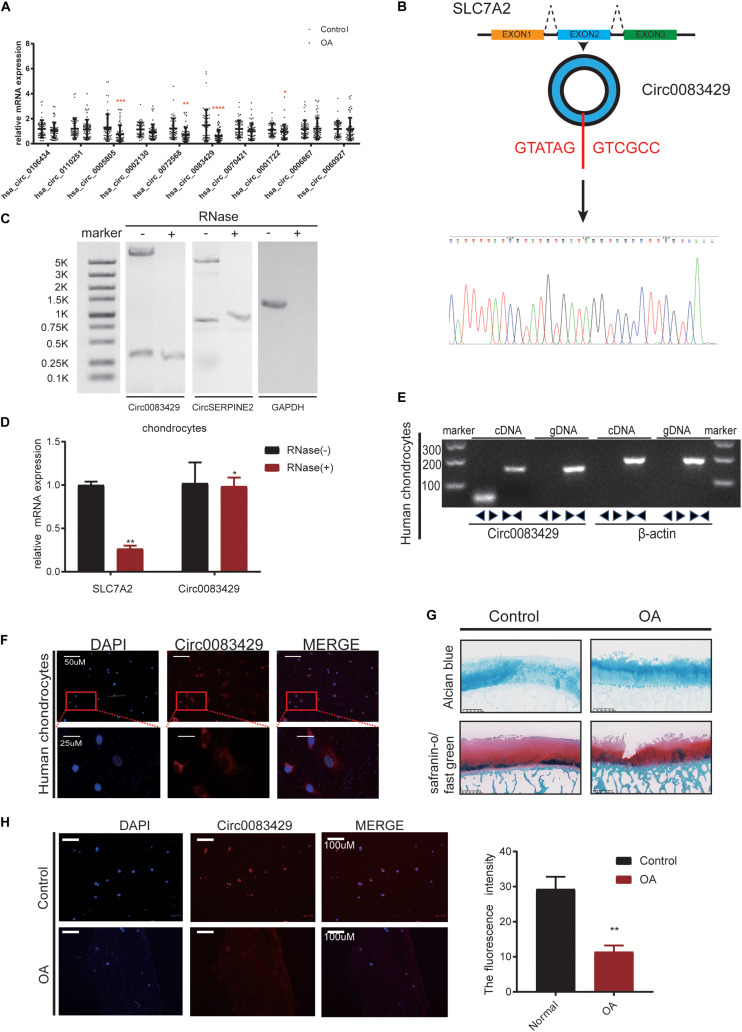
Osteoarthritis (OA) cartilage exhibits less Circ0083429 expression than that of non-OA cartilage. **(A)** Human OA (weight-bearing area) cartilage tissues exhibited less Circ0083429 expression than that of the human control (non-weight-bearing area) cartilage tissues. Data represent mean ± standard deviation (SD) (*n* = 20); *P < 0.05. **(B)** Schematic diagram showing that Circ0083429 (blue ring) is formed by the head-to-tail splicing of the second exon of the host *SLC7A2* gene. The results of Sanger sequencing confirmed the existence of Circ0083429. The red letters indicate the binding sites from head to tail. **(C)** After RNase treatment of total RNA, northern blot was performed to test the exist of Circ0083429 in chondrocytes, CircSERPINE2 (Shen et al., 2019) was used as the positive control, and GAPDH was used as negative control. **(D)** The expression of Circ0083429 and mRNA *SLC7A2* in chondrocytes was quantified by qRT-PCR after treatment with or without RNase R. Data represent the mean ± SD (*n* = 3); *P < 0.05. **(E)** DNA gel electrophoresis confirmed the existence of Circ0083429 by revealing that divergent primers can amplify Circ0083429 from cDNA, but not from genomic DNA in chondrocytes. Divergent primers cross through the splicing site of Circ0083429. Actin served as a positive control. **(F)** Fluorescence *in situ* hybridization (FISH) analysis revealed that Circ0083429 localized in the chondrocyte cytoplasm. Circ0083429 probes were labeled with Alexa Fluor 488, and nuclei were stained with 4,6-diamidino-2-phenylindole. **(G)** Alcian blue staining and safranin O/fast green staining of the cartilage from patients with weight-bearing and non-weight-bearing areas. Scale bar = 625 μm. **(H)** FISH analysis revealed that OA cartilage (weight-bearing area) tissues exhibited less Circ0083429 expression than the control cartilage (non-weight-bearing area) tissues. **p < 0.005.

We collected a total of 20 OA individuals, and the weight-bearing area and non-weight-bearing area of the 20 individuals are taken together for self-comparison. The non-weight bearing area served as the control group. It has been previously reported that circRNAs are differentially expressed in OA and non-OA cartilage (Shen et al., 2019). We used fold change as the screening standard to obtain the 10 circRNAs with the largest expression difference between up- and down-stream (Supplementary Table 1) from the National Center for Biotechnology Information (NCBI) Sequence Read Archive (SRA) database (SRA accession: PRJNA516555) (Shen et al., 2019). Of the 10 circRNAs, the difference in Circ0083429 expression was the most obvious in the 20 pairs of samples (Figure 1A). To prove the existence of Circ0083429, we designed a specific primer across the back-splicing site of the Circ0083429. Using this primer, we performed Sanger sequencing on the amplified DNA (Figure 1B), and the results showed that the amplified sequence contains the back-splicing sequence of Circ0083429 (GATATAGGTCGCC). Circ0083429 was subjected to RNase digestion to determine whether it was indeed a circRNA, and the stability of Circ0083429 was tested using Actinomycin D (Supplementary Figure 2A). Next, we used RNase R to digest total RNA, then performed northern blot, and found that Circ0083429 is more stable than its host gene mRNA *SLC7A2*, and the result demonstrated that Circ0083429 exists (Figure 1C). CircSERPINE2 (Shen et al., 2019) was used as positive control. After reverse transcription of the RNA following RNase treatment, qRT-PCR was performed after the amplification of Circ0083429 and its host gene *SLC7A2*. The result also proved that Circ0083429 was more stable than mRNA *SLC7A2* (Figure 1D). The primer of Circ0083429 is over the splicing site, while the primer of the linear gene *SLC7A2* is not over the splicing site. Genomic DNA (gDNA) and total RNA were extracted from human chondrocytes, and a band was detected by gel electrophoresis after reverse transcription of total RNA in chondrocytes. We found that the diverse primer of Circ0083429 can be amplified by cDNA but not gDNA, and the consequent primer of Circ0083429 can be amplified by both cDNA and gDNA; β-actin served as a positive control (Figure 1E). There is no reverse transcription product of Circ0083429 in gDNA, and the host gene *SLC7A2* of Circ0083429 is present in cDNA and gDNA, which further proves the characteristics of Circ0083429. This proved that Circ0083429 was formed by back-splicing. FISH revealed that Circ0083429 was primarily localized in the cytoplasm of human chondrocytes (Figure 1F). The weight-bearing area of the articular cartilage was used as OA, the non-weight-bearing area was used as the control, and then their degeneration was determined by safranin O/fast green and Alcian blue staining (Figure 1G). The control and OA cartilages were also subjected to FISH. The expression level of Circ0083429 in OA cartilage was lower than that in the adjacent control cartilage (Figure 1H). To ensure the abundance of Circ0083429, we determined the standard of Circ0083429 by absolute quantification (Supplementary Figure 2B). The above experiments proved that Circ0083429 exists in the cytoplasm and is differentially expressed in control tissues and OA.

### Circ0083429 Has a Protective Role in OA

**FIGURE 2:**
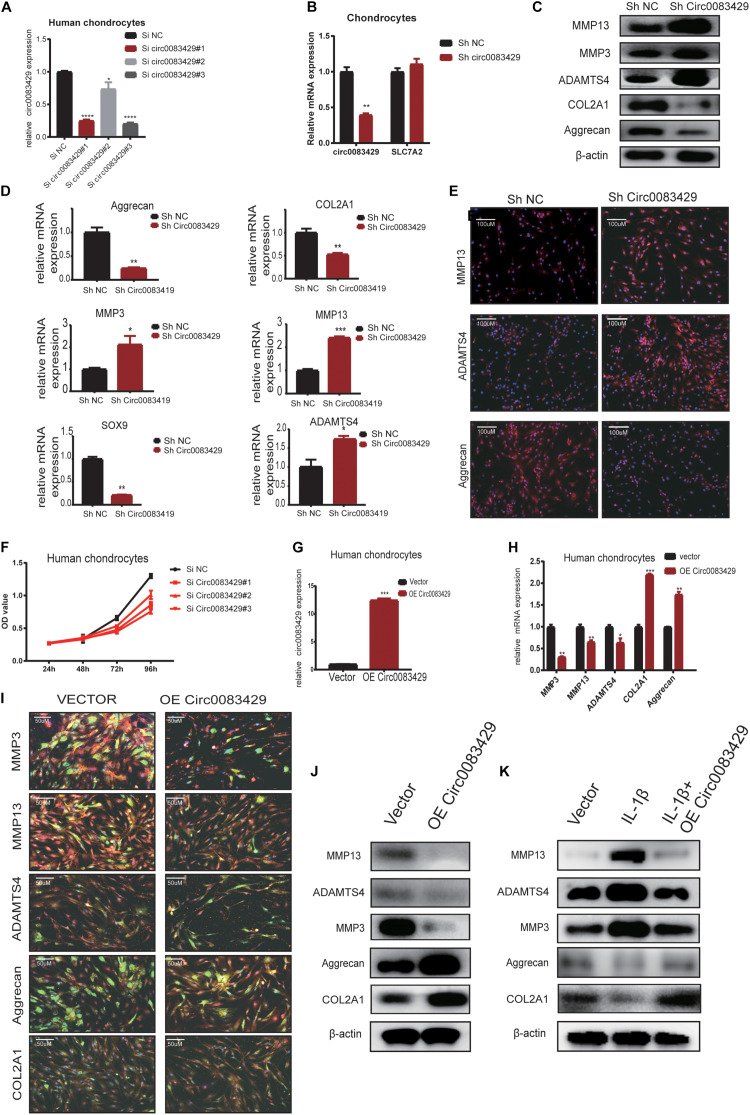
Knocking down Circ0083429 and overexpression of Circ0083429 regulates the levels of extracellular matrix enzymes in chondrocytes. **(A)** Circ0083429 siRNA or negative control siRNA (Si NC) was transfected into human chondrocytes at a final concentration of 20 nM. Transfection efficiency was measured by qRT-PCR 24 h after transfection. Expression was normalized to that of β-actin (*n* = 3); *P < 0.05. **(B)** After transfection with Circ0083429 short hairpin RNA (shRNA) for 24 h, Circ0083429 knockdown was confirmed, but the mRNA of Circ0083429 *SLC7A2* showed no change in expression. **(C)** Human chondrocytes were transfected with Circ0083429 shRNA or negative control (Sh-NC), and the mRNA expression of MMP3, MMP13, ADAMTS4, aggrecan, COL2A1, and SOX9 was measured by qRT-PCR (*n* = 3); *P < 0.05. **(D)** Human chondrocytes were transfected with Circ0083429 shRNA or sh-NC, and the protein levels of MMP13, MMP3, ADAMTS4, aggrecan, and COL2A1 were analyzed by western blot. **(E)** Expression levels of MMP13, ADAMTS4, and aggrecan were analyzed by immunofluorescence analysis after cells were transfected with Circ0083429 shRNA or sh-NC (scale bar = 100 μm). **(F)** Human chondrocytes were transfected with Circ0083429 siRNA or Si NC. Cell Counting Kit-8 was used to detect cell proliferation at 24, 48, 72, and 96 h (*n* = 3). **(G)** Human chondrocytes were transfected with Circ0083429 lentivirus plasmids or control plasmids (vector). Transfection efficiency was detected by qRT-PCR (*n* = 3); *P < 0.05. **(H)** Human chondrocytes were infected with Circ0083429 lentivirus or vector. Total RNA was extracted, and gene expression was quantified by qRT-PCR (*n* = 3); *P < 0.05. **(I,J)** Human chondrocytes were transfected with Circ0083429 lentivirus or vector, followed by western blot for MMP3, MMP13, ADAMTS4, COL2A1, and aggrecan, and immunofluorescence analysis for MMP3, MMP13, ADAMTS4, COL2A1, and aggrecan (scale bar = 50 μm) [green is green fluorescent protein (GFP) from the lentiviral vector]. **(K)** Human chondrocytes were co-transfected with Circ0083429 lentivirus after stimulation with IL-1β at a concentration of 10 ng/ml, and 48 h later, western blot was used to measure the expression of MMP13, MMP3, COL2A1, ADAMTS4, and aggrecan. **p < 0.005. ***p < 0.0005.

In order to explore the function of Circ0083429, stable knockdown and overexpression chondrocytes were constructed to investigate the effects of Circ0083429 on the ECM of chondrocytes. Three siRNAs against Circ0083429 were obtained from RiboBio and transfected into human chondrocytes. qRT-PCR revealed that one of the three siRNAs efficiently knocked down Circ0083429 (Figure 2A). Then, the SiRNA–Circ0083429#3 was used to construct shRNA. Transfection of the Circ0083429 shRNA did not affect the expression of *SLC7A2*, the mRNA target of Circ0083429 (Figure 2B). Upon Circ0083429 knockdown, the protein expression levels of MMP3, MMP13, and ADAMTS4 were upregulated, whereas those of COL2A1 and aggrecan were downregulated when compared with those of the negative control shRNA (Sh-NC) (Figure 2D). This indicated that Circ0083429 may have a protective role in OA. The results of western blot concurred with those of the qRT-PCR (Figure 2C). Immunofluorescence analysis of these proteins following Circ0083429 knockdown in human chondrocytes yielded results similar to those of the western blot and qRT-PCR analyses (Figure 2E). Furthermore, knockdown of Circ0083429 reduced the proliferative capacity of the human chondrocytes (Figure 2F).

We next constructed a Circ0083429 overexpression plasmid, transfected it into human chondrocytes, and confirmed that Circ0083429 was upregulated in the overexpression group (Figure 2G). Circ0083429 overexpression downregulated the expression of MMP3, MMP13, and ADAMTS4 and upregulated the expression of COL2A1 and aggrecan at the protein and mRNA levels as determined by western blot or qRT-PCR (Figures 2H–J). The protein levels were also observed by immunofluorescence analysis (Figure 2I). IL-1β is a cytokine known to promote OA (Goldring, 2000a; Daheshia and Yao, 2008). In order to detect whether overexpression of Circ0083429 could rescue the changes in inflammation indicators caused by IL-1β, we overexpressed Circ0083429 after IL-1β treatment. The results showed that protein changes regulated by IL-1β can be reversed by the overexpression of Circ0083429 in human chondrocytes (Figure 2K). Collectively, these data indicate that Circ0083429 played a significant role in the hemostasis of ECM by regulating the expression of catabolic enzymes and synthetases.

### Circ0083429 Functions in Osteoarthritis by Sponging MiR-346

**FIGURE 3:**
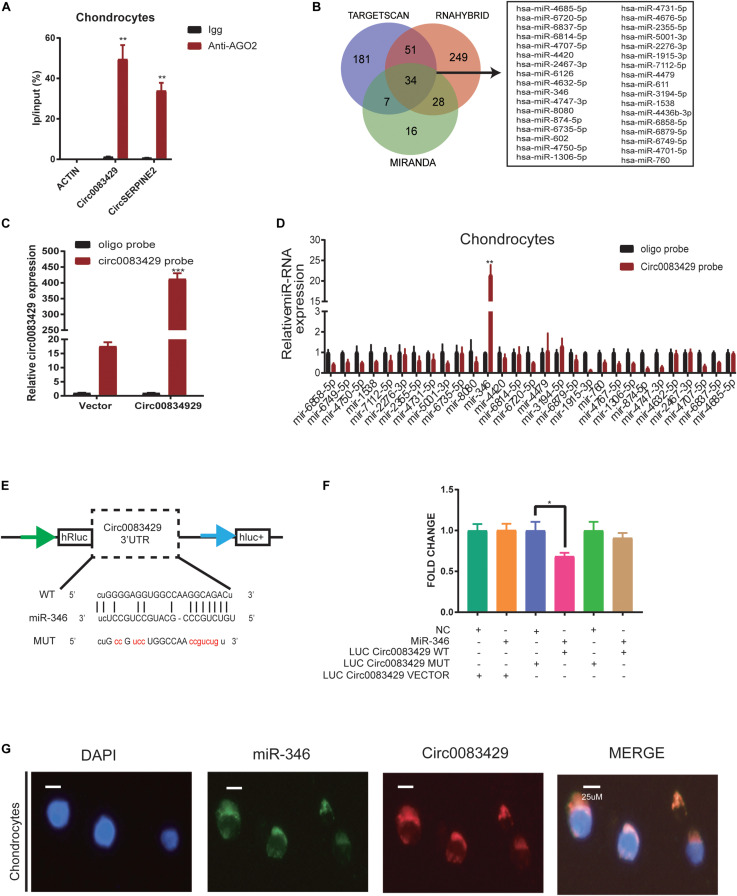
Circ0083429 sponges miR-346 to regulate the expression of matrix-degrading enzymes in human chondrocytes. **(A)** AGO2-RNA immunoprecipitation (RIP) assay was used to evaluate Circ0083429 and CircSERPINE2 levels in SW1353 cells, Igg was the negative control, and the CircSERPINE2 was positive control. Data represent mean ± SD (*n* = 3); *P < 0.05. **(B)** Three databases (miRanda, TargetScan, and RNAhybrid) predicted microRNAs that might bind Circ0083429, and the intersection of all databases was considered. miRanda screening criteria are Tot Score and Max Score >140, Tot Energy and Max Energy<–20 kcal/mol, TargetScan screening criteria is mef < –25 kcal/mol, and RNAhybrid screening criteria are the combining sites ≥ 1. **(C)** Lysates prepared from chondrocytes stably transfected with Circ0083429 or vector were subjected to qRT-PCR to test the efficiency of the transfection. Actin was used as a negative control. Data represent mean ± SD (*n* = 3); *P < 0.05. **(D)** The levels of 34 miRNAs predicted to bind Circ0083429 were quantified in the human chondrocyte lysates by qRT-PCR. **(E)** Binding site between Circ0083429 and miR-346 predicted by the starBase database. **(F)** Luciferase reporter assay was used to evaluate the luciferase activity of LUC–Circ0083429 WT and LUC–Circ0083429 mutant (MUT) in HEK-293T cells co-transfected with miR-346 mimic or mimic NC. Data represent mean ± SD (*n* = 3); *P < 0.05. The color code is as follows. Cyan, #009E73; orange, #F18F29; blue, #5B66AE; pink, #E63B86; green, #28AC38; dark yellow, #CCA162. **(G)** Fluorescence *in situ* hybridization (FISH) revealed the colocalization of Circ0083429 and miR-346 in human chondrocytes. Circ0083429 probes were labeled with Alexa Fluor 488, miR-346 probes were labeled with Cy3, and 4,6-diamidino-2-phenylindole was used to stain the nucleus (scale bar = 25 μm). **p < 0.005. ***p < 0.0005.

It has been reported that circRNAs function by sponging miRNAs (Hansen et al., 2013; Thomson and Dinger, 2016; Wang et al., 2016). CircRNA works through the sponge mechanism via the AGO2 protein. In order to study whether Circ0083429 works by this endogenous competition mechanism, we performed RIP and found that the *AGO2* protein could pull down Circ0083429 as compared with Igg, and CircSERPINE2 acted as a positive control (Figure 3A). To determine which miRNA–Circ0083429 binds to human chondrocytes, three databases (miRanda, TargetScan, and RNAhybrid) were used to predict the miRNAs that might bind Circ0083429, and the intersection of all databases was considered for further analyses. miRanda takes the Tot Score and Max Score > 140 and Tot Energy and Max Energy < −20 kcal/mol as the selective criteria, TargetScan takes mef < −25 kcal/mol as the selective criteria, and RNAhybrid takes the combine sites ≥ 1 as the standard. The results identified 34 candidate miRNAs (Figure 3B). We demonstrated that Circ0083429 could be enriched by Circ0083429 probe in Circ0083429-overexpressed chondrocytes more than that in WT chondrocytes, indicating the specificity of the probe (Figure 3C). In order to further confirm which miRNA that Circ0083429 binds to, we designed a biotin-labeled probe sequence and used the biotin-containing probe to perform RNA–RNA pulldown experiments in stably overexpressed Circ0083429 cell lines. And results proved that Circ0083429 and miR-346 had a strong correlation (Figure 3D). This result suggested that miR-346 possessed stronger ability to combine with Circ0083429 as directly compared with other miRNAs. Furthermore, the binding sequence was predicted using the starBase website^6^ (Figure 3E). To further confirm this combination of Circ0083429 and miR-346, a dual luciferase assay was performed. Luciferase activity was measured after cells were transfected with a miR-346 mimic or NC mimic and WT reporter or Mut reporter of Circ0083429. The results indicated that the luciferase activity was decreased when the cells were co-transfected with the WT reporter of Circ0083429 and miR-346 mimic (Figure 3F). In addition, RNA FISH indicated that Circ0083429 and miR-346 were colocalized in the chondrocyte cytoplasm (Figure 3G). These results demonstrated that miR-346 directly binds to Circ0083429 and may play a role in OA.

### MiR-346 Regulates the Hemostasis of Extracellular Matrix in Chondrocytes

**FIGURE 4:**
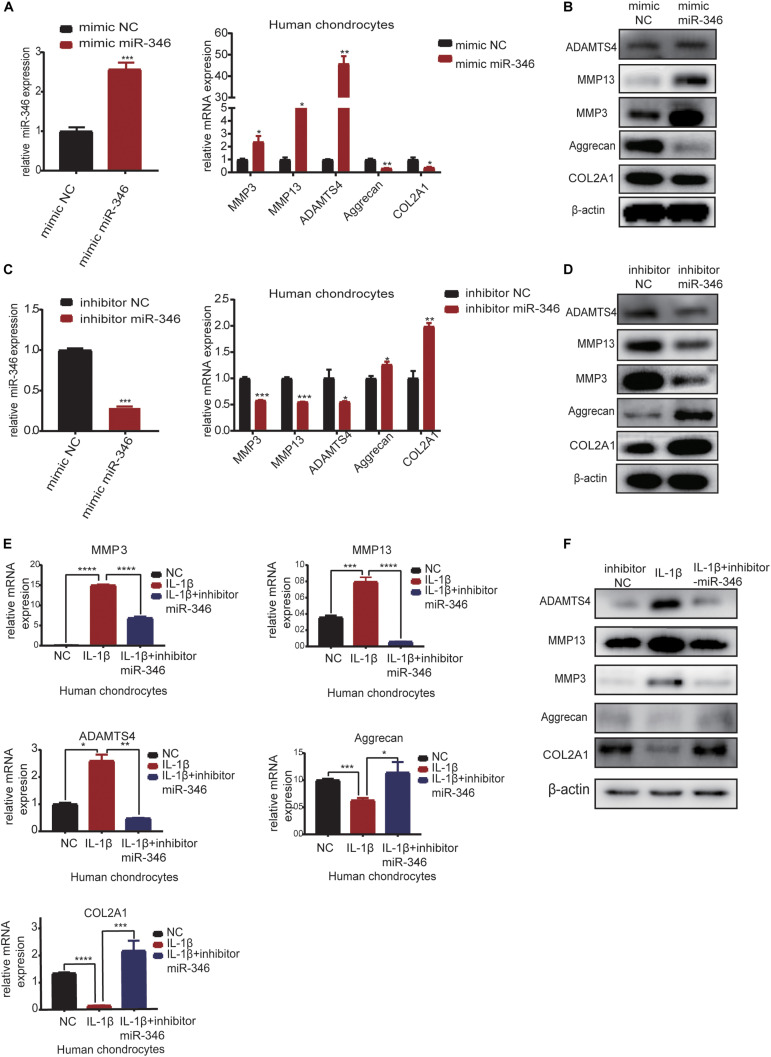
MiR-346 promotes the progression of osteoarthritis (OA) and miR-346 inhibition mitigates IL-1β-induced inflammation. **(A)** MiR-346 expression and MMP3, MMP13, ADAMTS4, COL2A1, and aggrecan mRNA expression in chondrocytes was quantified by qRT-PCR 24 h after transfection with miR-346 mimic or mimic NC at a final concentration of 20 nM (*n* = 3); *P < 0.05. **(B)** MMP3, MMP13, ADAMTS4, COL2A1, and aggrecan protein expression in chondrocytes was measured by western blot 48 h after transfection with miR-346 mimic or mimic NC at a final concentration of 20 nM (*n* = 3); *P < 0.05. **(C)** MiR-346 expression and MMP3, MMP13, ADAMTS4, COL2A1, and aggrecan mRNA expression in chondrocytes was quantified by qRT-PCR 24 h after transfection with miR-346 inhibitor or inhibitor NC at a final concentration of 20 nM (*n* = 3); *P < 0.05. **(D)** MMP3, MMP13, ADAMTS4, COL2A1, and aggrecan protein expression in chondrocytes was measured by western blot 48 h after transfection with miR-346 inhibitor or inhibitor NC at a final concentration of 20 nM (*n* = 3); *P < 0.05. **(E)** The mRNA levels of related genes were measured by qRT-PCR after treatment with IL-1β at a concentration of 10 ng/ml, or IL-1β with a concentration of 10 ng/ml, and miR-346 inhibitor at a final concentration of 20 nM for 24 h (*n* = 3) *P < 0.05. **(F)** The protein levels of related genes were measured by western blot after treatment with IL-1β at a concentration of 10 ng/ml, or IL-1β with a concentration of 10 ng/ml, and miR-346 inhibitor at a final concentration of 20 nM for 48 h (*n* = 3) *P < 0.05. **p < 0.005. ***p < 0.0005. ****p < 0.00005.

In order to explore the function of miR-346 in chondrocytes, we transfected the mimic miR-346 into human chondrocytes with a corresponding mimic NC to a final concentration of 20 nM. After 24 h, RNA was extracted from the mimic-transfected cells and subjected to qRT-PCR analysis. The results revealed that miR-346 expression was higher in the miR-346 mimic-transfected cells and lower in the miR-346 inhibitor-transfected cells (Figure 4A). qRT-PCR analysis revealed that the mRNA expression levels of MMP3, MMP13, and ADAMTS4 were upregulated, whereas those of COL2A1 and aggrecan were downregulated in the miR-346 mimic-transfected chondrocytes. After 48 h from the transfection of the miR-346 mimic into the human chondrocytes, total protein was extracted and western blot was performed to test the expression of these genes. The protein levels were comparable with the mRNA levels (Figure 4B). To further study the effects of miR-346 on chondrocytes, we transfected chondrocytes with inhibitor miR-346 or inhibitor NC. The RNA was extracted 24 h after transfection; the expression of miR-346 was downregulated along with MMP3, MMP13, and ADAMTS4; and COL2A1 and aggrecan were upregulated (Figure 4C). The protein was extracted 48 h after transfection with the miR-346 inhibitor. Further, western blot showed results opposite to those of the transfection with the miR-346 mimic (Figure 4D). IL-1β, a pro-inflammatory factor, can induce inflammation (Goldring, 2000a; Daheshia and Yao, 2008). To determine whether the inflammation inhibitor miR-346 can reverse the inflammatory activation of chondrocytes by IL-1β, we co-transfected IL-1β and the miR-346 inhibitor into human chondrocytes, which reversed the IL-1β-induced inflammation in the chondrocytes at the mRNA and protein levels (Figures 4E,F). These results indicate that miR-346 can promote the progression of inflammation and that inhibiting miR-346 can alleviate the inflammation induced by IL-1β.

### MiR-346 Functions in Human Cartilage by Binding the Downstream Target Gene *SMAD* Family Member 3 (*SMAD3*)

**FIGURE 5:**
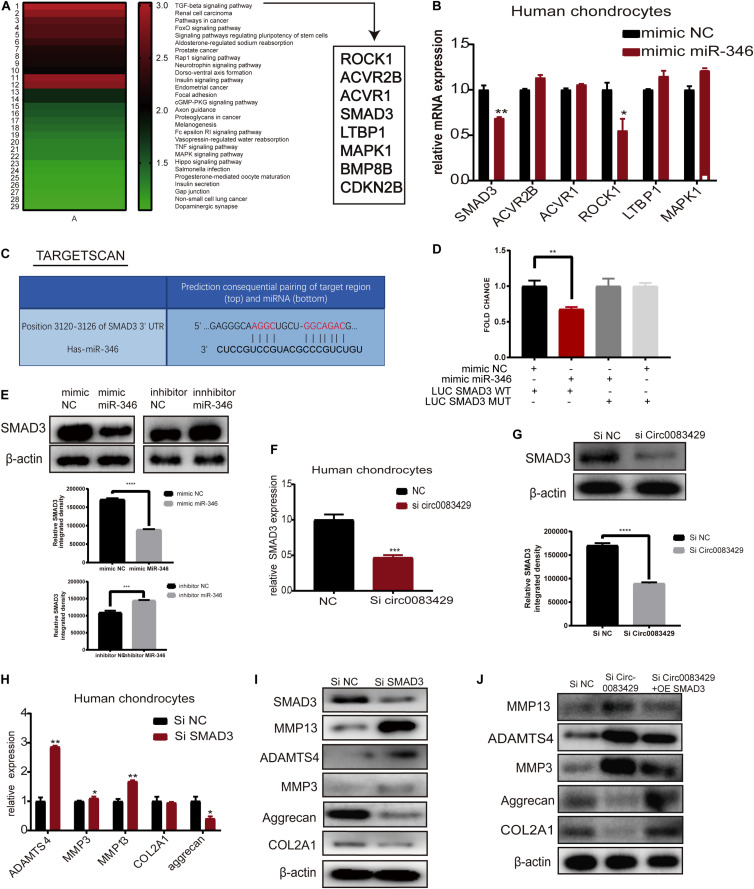
MiR-346 binds *SMAD3* in chondrocytes. **(A)** The Kyoto Encyclopedia of Genes and Genomes pathway analysis was performed using the Database for Annotation, Visualization, and Integrated Discovery with 382 genes predicted to bind miR-346 by *miRDIP*. **(B)** Eight genes associated with miR-346 were enriched in the TGF-β pathway. The expression levels of these eight genes were quantitatively analyzed by qRT-PCR after the chondrocytes were transfected with miR-346 mimic NC for 24 h. Data represent mean ± SD (*n* = 3); *P < 0.05. **(C)** TargetScan predicts the binding sites of miR-346 and *SMAD3*. **(D)** Luciferase reporter assay was performed to test the luciferase activity of LUC-*SMAD3* or LUC-*SMAD3* mutant co-transfected with miR-346 mimic or NC mimic in HEK-293T cells (*n* = 3); *P < 0.05. Black, mimic NC + LUC SMAD3 WT group; red, mimic miR-346 + LUC SMAD3 WT group; dark gray, mimic miR-346 + LUC SMAD3 MUT group; light gray, mimic NC + LUC SMAD3 MUT group. **(E)** *SMAD3* protein levels were analyzed after transfection with miR-346 mimic or miR-346 inhibitor at a concentration of 20 nM as compared with mimic NC or inhibitor NC for 48 h. **(F,G)** The mRNA expression levels were detected in Circ0083429 siRNA-transfected chondrocytes or Si NC transfected chondrocytes at a concentration of 20 nM for 24 h by qRT-PCR; the protein expression levels were detected in Circ0083429 siRNA-transfected chondrocytes or Si NC transfected chondrocytes at a concentration of 20 nM for 48 h by western blot; *P < 0.05. **(H,I)** Human chondrocytes were transfected with SMAD3 siRNA at a final concentration of 20 nM. After 24 h of transfection, the mRNA expression levels of related genes were detected in the chondrocytes by qRT-PCR. Data represent mean ± SD (*n* = 3); *P < 0.05. After 48 h, the protein expression levels of SMAD3, COL2A1, MMP13, MMP3, ADAMTS4, and aggrecan were detected by western blot. **(J)** After Si Circ0083429 at a concentration of 20 nm, co-transfected *SMAD3* plasmids, and we observe the protein expression of related genes in chondrocytes by western blot for 48 h of transfection. **p < 0.005. ***p < 0.0005. ****p < 0.00005.

In miRDIP, we predicted the target genes bound downstream of miR-346, and a total of 19,636 predicted bound target genes were found. We used the score class to screen for the top 1% with the highest score and obtained 382 genes. We then used the DAVID database to perform KEGG analysis on these 382 genes (Supplementary Table 2). KEGG analysis identified 29 signaling pathways related to these genes, with the most relevant being the TGF-β signaling pathway (Figure 5A). The mimic miR-346 was transfected into human chondrocytes to analyze changes in eight genes, ROCK1, ACVR2B, ACVR1, SMAD3, LTBP1, MAPK1, BMP8B, and CDKN2B, that are involved in the TGF-β pathway. The mRNA expression of *SMAD3* and *ROCK1* in human chondrocytes was markedly downregulated upon transfection with the miR-346 mimic compared with other genes (Figure 5B). A previous study demonstrated that *SMAD3* regulates the inflammation during development and progression in OA (Wang et al., 2018). Therefore, we chose *SMAD3* for downstream research. Additionally, there were two genes, *BMP8P* and *CDKN2B*, with expression levels too low for analysis. In TargetScan, we identified the miR-346 binding sites in *SMAD3* (Figure 5C). To further confirm the combination of miR-346 and *SMAD3*, the mimic miR-346 was transfected into HEK-293T cells, and luciferase activity was measured by co-transfection with a WT reporter or Mut reporter of *SMAD3*. Luciferase activity of the WT reporter was decreased compared with that of the Mut reporter (Figure 5D). The pattern of sequence of a WT reporter or Mut reporter of *SMAD3* combined with miR-346 is in Supplementary Figure 4A. *SMAD3* protein expression was regulated by miR-346 in human chondrocytes (Figure 5E). *SMAD3* has been reported to play a role in inflammation (Chen et al., 2019). Several studies have demonstrated that mutations in *SMAD3* cause multiple aneurysms in patients without OA symptoms (Aref-Eshghi et al., 2014). Thus, it was speculated that Circ0083429 exhibits a protective effect in OA via the *SMAD3* pathway. qRT-PCR and western blot analyses revealed that the mRNA and protein expression levels of *SMAD3* were downregulated upon Circ0083429 knockdown in human chondrocytes compared with those of the control (Figures 5F,G). Further, the function of *SMAD3* was analyzed by transfecting human chondrocytes with siRNA against *SMAD3* (si*SMAD3*). In si*SMAD3*-transfected human chondrocytes, the mRNA and protein expression levels of MMP3, MMP13, and ADAMTS4 were upregulated, whereas those of COL2A1 and aggrecan were downregulated (Figures 5H,I). Most interestingly, the changes in protein expression associated with Circ0083429 knockdown were reversed by the overexpression of *SMAD3* (Figure 5J). These results suggest that Circ0083429 regulates OA by influencing the downstream target genes of *SMAD3*.

### Circ0083429 Has a Protective Effect in OA *in vivo*

**FIGURE 6:**
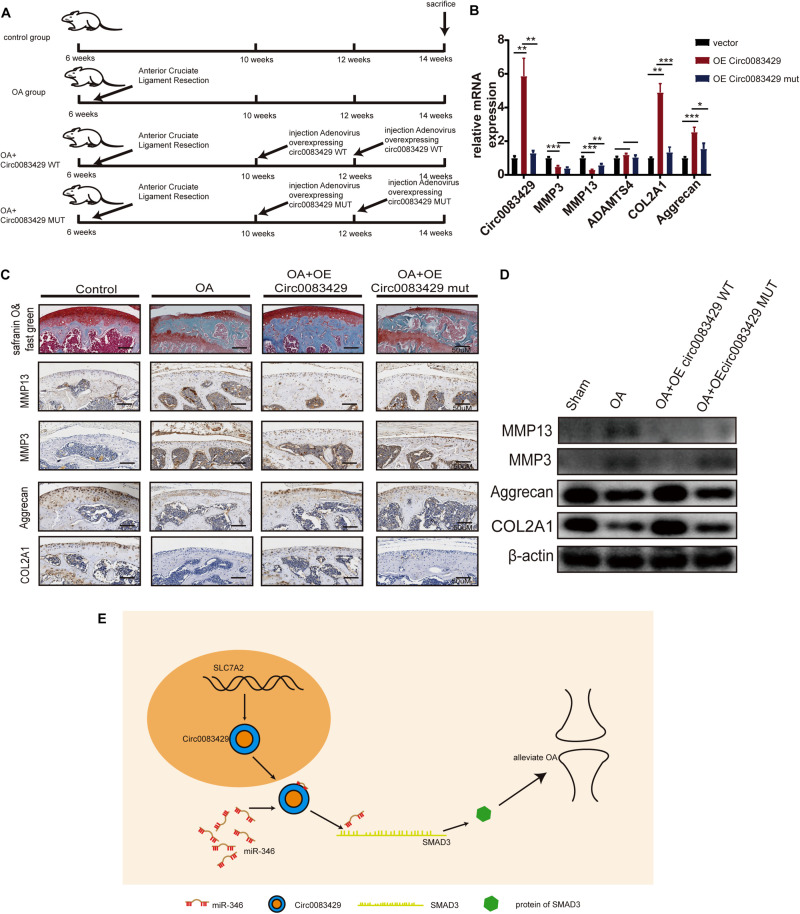
Circ0083429 alleviates osteoarthritis (OA) in a mouse model of OA. **(A)** Schematic diagram of the animal experiment. **(B)** The mRNA levels of MMP3, MMP13, ACOL2A1, and aggrecan in mouse cartilage tissues were detected and qRT-PCR (*n* = 3); *P < 0.05. **(C)** Safranin O/fast green staining and immunohistochemistry of MMP3, MMP13, aggrecan, and COL2A1 of mouse cartilage were performed when mice were sacrificed (scale bar = 50 μm). **(D)** Protein expression of MMP3, MMP13, ACOL2A1, and aggrecan in mouse cartilage tissues was detected by western blot (*n* = 3); *P < 0.05. **(E)** Schematic of the work. Dark yellow, nucleus; blue, Circ0083429; red, MiR-346; grass green, mRNA of SMAD3; green, protein of SMAD3. **p < 0.005. ***p < 0.0005.

To explore the effect of Circ0083429 on OA *in vivo*, we divided 24 C57BL mice into four groups, each with six mice. At 6 weeks of age, the mice underwent anterior cruciate ligament resection surgery on both knee joints for the establishment of OA models. After 4 weeks, at 10 weeks of age, two of the surgery groups were selected to be injected with AAV Circ0083429 overexpression (WT) or Circ0083429 Mut overexpression (MUT) and injected with the AAV Circ0083429 overexpression (WT) or Circ0083429 Mut overexpression (MUT) again when mice were 12 weeks old. Finally, the mice were sacrificed at 14 weeks (Figure 6A). The tissue protein and tissue RNA of the knee joint were extracted to test the protein and mRNA levels (Figures 6B,D). The results demonstrated that the expression Circ0083429 was upregulated after injection with the AAV Circ0083429 overexpression (WT). We then used the right knee joint tissue to collect tissue sections and subjected them to safranin O/fast green staining and immunohistochemistry (Figure 6C). The results suggest that Circ0083429 overexpression (WT) could alleviate OA, but Circ0083429 Mut overexpression (MUT) could not. Altogether, these findings suggested that Circ0083429 regulated the expression of *SMAD3* by sponging miR-346 and furtherly affected the progression of OA by regulating the hemostasis of ECM in chondrocytes (Figure 6E).

## Discussion

At present, treatment options for OA are inadequate (Wieland et al., 2005; Sinusas, 2012; Lane et al., 2017). Therefore, it is necessary to conduct further research on the occurrence and development of OA. A previous study found that CircSERPINE2 alleviates OA by targeting miR-1261 (Shen et al., 2019), which means that circRNA plays a regulatory role in the occurrence and development of OA.

Here, we identified a novel circRNA, Circ0083429, which was generated by the back-splicing of the second exon of the *SLC7A2* gene. Circ0083429 expression was lower in the OA tissue than in the control tissue. By knocking down or overexpressing Circ0083429 in chondrocytes, we demonstrated that Circ0083429 protects the ECM from degradation. This suggests that Circ0083429 has a function in OA and may act as an underlying target for OA treatment. To further study the role of Circ0083429 in OA, we identified the downstream target of Circ0083429, miR-346, which has been reported to promote hepatocellular carcinoma progression by regulating the expression of breast cancer metastasis suppressor 1 (Guo et al., 2018). However, the relationship between miR-346 and OA has not been investigated previously. In this study, we found that miR-346 can downregulate the expression of synthetases and upregulate the expression of degrading enzymes and that the inhibition of miR-346 plays an opposite role. Previous studies reported that miRNAs regulated the progression of OA and act as a target for OA treatment (Si et al., 2017; Lian et al., 2018; Cheleschi et al., 2019; Nakamura et al., 2019; Wu et al., 2019). Here, we provide a new target for the treatment of OA.

In order to explore the mechanism of effect of Circ0083429 on OA, we introduced a cytokine, IL-1β.

IL-1β belongs to the IL-1 family and is an important inflammatory factor that triggers the apoptosis of chondrocytes and promotes the progression of OA (Lopez-Armada et al., 2006; Hasko and Cronstein, 2013). Here, we used IL-1β to stimulate chondrocytes and found that OA-related synthetases were reduced while degrading enzymes were increased. Overexpression of Circ0083429 or inhibition miR-346 could rescue these changes. This demonstrated that the upregulation of Circ0083429 or downregulation of miR-346 can protect the ECM from IL-β-induced degradation. To explore the downstream of miR-346, subsequent analyses revealed that miR-346 repressed *SMAD3* expression. *SMAD3*, member 3 of the *SMAD* family, consists of at least eight different SMADS in humans. A previous study demonstrated that *SMAD3*-deficit mice induced the hypertrophy of chondrocytes and stimulated the osteophyte formation. The mutation of *SMAD3* is associated with OA in human (Yang et al., 2001; Yao et al., 2003). Meta-analyses have indicated that *SMAD3* rs12901499 polymorphism increases the risk of OA (Yang et al., 2018). However, the regulation of *SMAD3* by Circ0083429 to regulate OA is unknown. In this study, we clarified that the expression levels of *SMAD3* can be regulated by Circ0083429 and that the overexpression of *SMAD3* can rescue the protein changes brought about by Circ0083429.

In the animal experiments, we made OA model by anterior cruciate ligament resection surgery. Safranin O/fast green verifies the success of this modeling. To verify the role of Circ0083429 *in vitro*, we injected adenoviruses overexpressing Circ0083429 (WT) into the joints of mice after surgery. Four weeks later, we found that injecting overexpressing Circ0083429 (WT) could delay the progression of OA, but adenoviruses overexpressing Mut Circ0083429 (MUT) could not. This demonstrated that Circ0083429 can alleviate the progression of OA in mice.

It is still possible that Circ0083429 could bind other target genes to regulate OA. However, we determined that Circ0083429 can regulate the expression of the ECM of chondrocytes by regulating the expression of downstream miR-346/SMAD3.

## Conclusion

In this study, we identified a new circRNA, Circ0083429, that exhibits lower expression levels in OA tissue and can regulate OA by sponging downstream miR-346/*SMAD3*. The Circ0083429/miR-346/*SMAD3* axis can regulate the key proteins MMP3, MMP13, ADAMTS4, aggrecan, and COL2A1 in OA. *In vivo*, Circ0083429 overexpression could alleviate OA as compared with the untreated group. The findings of this study thus present Circ0083429 or miR-346 as a potential therapeutic target for OA and provide a basis for the further elucidation of the role of Circ0083429 in OA.

## Data Availability Statement

The raw data supporting the conclusions of this article will be made available by the authors, without undue reservation, to any qualified researcher.

## Ethics Statement

The studies involving human participants were reviewed and approved by Ethics Committee of Sir Run Run Shaw Hospital. The patients/participants provided their written informed consent to participate in this study. The animal study was reviewed and approved by Ethics Committee of Sir Run Run Shaw Hospital. Written informed consent was obtained from the individual(s) for the publication of any potentially identifiable images or data included in this article.

## Author Contributions

JM, SF, and TY designed the experiments. TY, YY, and ZX performed the experiments and acquired the data. TY, YX, YH, JG, SS, HY, and YI analyzed the data. JM, SF, and TY supervised the project and wrote the manuscript. All authors contributed to the article and approved the submitted version.

## Conflict of Interest

The authors declare that the research was conducted in the absence of any commercial or financial relationships that could be construed as a potential conflict of interest.

## Footnotes

1 http://www.miranda.org

2 http://bibiserv.techfak.uni-bielefeld.de/rnahybrid

3 http://www.targetscan.org/

4 http://ophid.utoronto.ca/mirDIP/index.jsp

5 https://david.ncifcrf.gov/summary.jsp

6 http://starbase.sysu.edu.cn/
