# Supplementary material for: Circ0083429 Regulates Osteoarthritis Progression via the Mir-346/SMAD3 Axis
Source: Front Cell Dev Biol. 2021 Jan 15;8:579945. doi: 10.3389/fcell.2020.579945 (PMC7843588; doi:10.3389/fcell.2020.579945)
Supplement: Supplementary file 1 [file Table_1.DOCX]

| Supplementary Table S1 | | | | | |
| --- | --- | --- | --- | --- | --- |
| id | log2FC(OA/Control) | pval | fdr | regulate | significant |
| 1:102979382\|102989571 | -7.0146886 | 9.33E-11 | 1.13E-06 | down | yes |
| 17:27765535\|27766588 | -7.2095933 | 2.94E-10 | 1.78E-06 | down | yes |
| 19:6702127\|6702579 | -5.7256104 | 3.17E-08 | 0.00012766 | down | yes |
| 5:59180595\|59215968 | -5.2760784 | 1.18E-07 | 0.00035593 | down | yes |
| 8:17558295\|17562110 | -5.830035 | 7.60E-07 | 0.00183471 | down | yes |
| 7:90747681\|90790652 | -4.4750758 | 3.16E-06 | 0.00636129 | down | yes |
| 4:88475841\|88479507 | -4.5431287 | 4.12E-06 | 0.00710284 | down | yes |
| 13:75713182\|75727098 | 4.06243391 | 4.95E-06 | 0.00748029 | up | yes |
| 17:27778690\|27779056 | -4.2259783 | 1.16E-05 | 0.01510205 | down | yes |
| 4:150467673\|150491035 | -4.2555494 | 1.27E-05 | 0.01510205 | down | yes |
| 8:17543318\|17543715 | -4.576294 | 1.42E-05 | 0.01510205 | down | yes |
| 10:115120185\|115129535 | 3.52956202 | 1.50E-05 | 0.01510205 | up | yes |
| 5:65170617\|65215487 | -3.9400185 | 6.71E-05 | 0.06130968 | down | yes |
| 8:41661430\|41661941 | 3.31749642 | 7.68E-05 | 0.06130968 | up | yes |
| 1:247159006\|247159813 | 5.42175065 | 8.25E-05 | 0.06130968 | up | yes |
| 20:54157169\|54171670 | -4.2436025 | 8.27E-05 | 0.06130968 | down | yes |
| 1:46077515\|46080750 | -3.9505609 | 8.63E-05 | 0.06130968 | down | yes |
| 2:223912253\|223918010 | -4.0531467 | 0.00013432 | 0.0878503 | down | yes |
| 15:83923904\|83970637 | -3.7749837 | 0.0001382 | 0.0878503 | down | yes |
| 17:27768977\|27769584 | -5.1367225 | 0.0001862 | 0.11244761 | down | yes |
| 19:6697344\|6697794 | -3.5244413 | 0.00030733 | 0.17675827 | down | yes |
| 7:84110470\|84194668 | -4.3614667 | 0.00034832 | 0.19122922 | down | yes |
| 20:45815541\|45815913 | -3.6184204 | 0.00038076 | 0.1999479 | down | yes |
| 7:84129123\|84194668 | -4.4164234 | 0.00040484 | 0.20373628 | down | yes |
| 1:246591512\|246591941 | 3.25903817 | 0.00043672 | 0.21098767 | up | yes |
| 19:48845370\|48852326 | -3.44724 | 0.00059645 | 0.2705299 | down | yes |
| 6:159682474\|159688242 | -3.2773535 | 0.00060476 | 0.2705299 | down | yes |
| 21:41432050\|41437152 | -3.5076824 | 0.00084407 | 0.36409636 | down | yes |
| 12:100204940\|100205744 | -3.4815857 | 0.0009696 | 0.37359312 | down | yes |
| 7:84046324\|84110589 | -3.4648439 | 0.00097324 | 0.37359312 | down | yes |
| 2:72674532\|72733118 | 4.56218595 | 0.00098611 | 0.37359312 | up | yes |
| 1:93305388\|93325895 | -3.4219483 | 0.00098981 | 0.37359312 | down | yes |
| 1:27668226\|27669346 | -3.85047 | 0.00106467 | 0.38966852 | down | yes |
| 1:23071225\|23072197 | 3.52357915 | 0.00111472 | 0.39598924 | up | yes |
| 15:45476079\|45476552 | -3.9598305 | 0.00122753 | 0.42360477 | down | yes |
| 12:27650010\|27656730 | -3.3147103 | 0.00128896 | 0.42516483 | down | yes |
| 17:66513336\|66514466 | -4.3901789 | 0.00130246 | 0.42516483 | down | yes |
| 5:138424075\|138425582 | -3.355593 | 0.00142197 | 0.4519615 | down | yes |
| 22:33761371\|33765953 | -4.1946536 | 0.00159529 | 0.47739629 | down | yes |
| 12:100204940\|100205757 | -3.3785056 | 0.00159788 | 0.47739629 | down | yes |
| 5:38971877\|38978650 | -3.191256 | 0.00162057 | 0.47739629 | down | yes |
| 3:10226371\|10239039 | -3.2309863 | 0.00166474 | 0.47873048 | down | yes |
| 6:168543625\|168608239 | 4.56983277 | 0.00170875 | 0.4799612 | up | yes |
| 21:36338779\|36344707 | 4.11644082 | 0.00199654 | 0.54804915 | up | yes |
| 20:18297985\|18306393 | -4.6873711 | 0.00214476 | 0.57565226 | down | yes |
| 11:63895159\|63895633 | 2.51804111 | 0.00221361 | 0.58121793 | up | yes |
| 17:80287815\|80291827 | -3.0144506 | 0.00280045 | 0.71965506 | down | yes |
| 7:84110470\|84134951 | -3.4826538 | 0.00296286 | 0.73284844 | down | yes |
| 20:25496663\|25498346 | 2.04618052 | 0.00300782 | 0.73284844 | up | yes |
| 11:92352096\|92355404 | 4.16268873 | 0.00303382 | 0.73284844 | up | yes |
| 11:78119182\|78121174 | 3.15142761 | 0.00315668 | 0.73805944 | up | yes |
| 1:236859833\|236863554 | -3.1032671 | 0.00317903 | 0.73805944 | down | yes |
| 12:121023990\|121027817 | -3.0764678 | 0.00326587 | 0.73805944 | down | yes |
| 4:102304317\|102315830 | -3.8014826 | 0.00336046 | 0.73805944 | down | yes |
| 9:84702159\|84710791 | 2.86567306 | 0.00336093 | 0.73805944 | up | yes |
| 18:6263947\|6312056 | -3.4977975 | 0.00378226 | 0.80234344 | down | yes |
| 3:112109543\|112113145 | -3.3472394 | 0.00378652 | 0.80234344 | down | yes |
| 7:72890627\|72891110 | -3.6844846 | 0.00398994 | 0.83086978 | down | yes |
| 1:32944019\|32949774 | -2.9216687 | 0.00524299 | 1 | down | yes |
| 11:125235045\|125239728 | -3.3103606 | 0.00537553 | 1 | down | yes |
| 9:33935839\|33941862 | -3.5440215 | 0.00559316 | 1 | down | yes |
| 22:33761371\|33856802 | -3.727331 | 0.0056138 | 1 | down | yes |
| 11:16183886\|16234671 | -2.8317215 | 0.00570934 | 1 | down | yes |
| 4:186706563\|186709845 | 4.04786425 | 0.00589761 | 1 | up | yes |
| 3:125277726\|125331238 | 3.80512692 | 0.0061645 | 1 | up | yes |
| 4:102304317\|102315742 | -2.9703422 | 0.00619047 | 1 | down | yes |
| 17:80325030\|80328477 | -3.013455 | 0.00630213 | 1 | down | yes |
| 19:45022053\|45028992 | -3.2840934 | 0.00675664 | 1 | down | yes |
| 16:58560212\|58560362 | 3.91773309 | 0.00679791 | 1 | up | yes |
| 4:106295094\|106308989 | -2.8955174 | 0.00705903 | 1 | down | yes |
| 10:32805015\|32824604 | 2.47034385 | 0.00779452 | 1 | up | yes |
| 12:121027576\|121027817 | -2.8648954 | 0.00811642 | 1 | down | yes |
| 20:35069296\|35078174 | -2.730087 | 0.00862442 | 1 | down | yes |
| 7:98228014\|98229166 | 3.61267813 | 0.00873229 | 1 | up | yes |
| 1:186893011\|186911389 | -3.1066736 | 0.00897813 | 1 | down | yes |
| 20:38040435\|38066256 | 2.91024396 | 0.00929394 | 1 | up | yes |
| 2:241030284\|241030571 | 2.3421537 | 0.00954736 | 1 | up | yes |
| 5:175492095\|175492267 | 3.5921128 | 0.00962401 | 1 | up | yes |
| 13:42953948\|42970670 | -2.9075567 | 0.00963706 | 1 | down | yes |
| 15:72695177\|72716850 | -2.8364617 | 0.00965302 | 1 | down | yes |
| 5:65260600\|65300131 | -2.7507601 | 0.00967631 | 1 | down | yes |
| 9:120536372\|120539164 | 2.2484411 | 0.00992009 | 1 | up | yes |
| 17:80306252\|80306468 | -2.7923131 | 0.01004225 | 1 | down | yes |
| 12:23604387\|23741039 | -2.7154978 | 0.01020717 | 1 | down | yes |
| 7:80789306\|80800826 | -2.8798374 | 0.01077985 | 1 | down | yes |
| 5:72877228\|72883232 | 2.0345252 | 0.01078439 | 1 | up | yes |
| 17:1480527\|1480885 | 1.65920685 | 0.01114306 | 1 | up | yes |
| 12:25995037\|26003450 | -3.4698886 | 0.01185674 | 1 | down | yes |
| 13:42917541\|42970670 | -3.0941484 | 0.01194371 | 1 | down | yes |
| 1:21861757\|21862115 | 1.9291177 | 0.01207133 | 1 | up | yes |
| 3:100841998\|100848875 | -2.6548355 | 0.01233469 | 1 | down | yes |
| 6:168509915\|168608239 | 3.54082201 | 0.01235108 | 1 | up | yes |
| 11:125602303\|125604247 | -2.6103884 | 0.01252699 | 1 | down | yes |
| 7:84046324\|84060558 | -4.0256687 | 0.0126723 | 1 | down | yes |
| 7:157366502\|157367483 | 2.1840816 | 0.01295983 | 1 | up | yes |
| 2:223991803\|224001922 | -2.3340353 | 0.01316184 | 1 | down | yes |
| 5:139614586\|139623461 | -2.6963238 | 0.0134045 | 1 | down | yes |
| 1:65913245\|65918835 | -2.298018 | 0.01342013 | 1 | down | yes |
| 19:45011936\|45012276 | -2.5997947 | 0.01370568 | 1 | down | yes |
| 5:618990\|620261 | -2.7551739 | 0.0139525 | 1 | down | yes |
| 2:237347807\|237353403 | 3.37387478 | 0.01416384 | 1 | up | yes |
| 11:74118228\|74118382 | -2.7478754 | 0.01430979 | 1 | down | yes |
| 19:45025329\|45025737 | -2.6678942 | 0.0143445 | 1 | down | yes |
| 1:10403071\|10404279 | 2.33188823 | 0.0146809 | 1 | up | yes |
| 7:90726567\|90863269 | -2.5316566 | 0.01468444 | 1 | down | yes |
| 5:65170617\|65197151 | -4.1541297 | 0.01529692 | 1 | down | yes |
| 10:26934413\|26935627 | -3.0092422 | 0.01538201 | 1 | down | yes |
| 18:54277703\|54287411 | 3.36487091 | 0.01564169 | 1 | up | yes |
| 20:64022757\|64026058 | 2.15899955 | 0.01569604 | 1 | up | yes |
| 12:23563258\|23575838 | -2.7800811 | 0.01604729 | 1 | down | yes |
| 5:32724698\|32739030 | 2.31448473 | 0.01628771 | 1 | up | yes |
| 4:88475841\|88494331 | -2.9231002 | 0.01870983 | 1 | down | yes |
| 10:74589282\|74600493 | -2.8672356 | 0.01990161 | 1 | down | yes |
| 3:119843255\|119876508 | -2.4410486 | 0.02001182 | 1 | down | yes |
| 9:91756071\|91775818 | -2.5418219 | 0.02001683 | 1 | down | yes |
| 22:38521608\|38568289 | 3.30156947 | 0.02035607 | 1 | up | yes |
| 11:74132844\|74133557 | -2.6353688 | 0.02039675 | 1 | down | yes |
| 12:108652272\|108701323 | 1.8429984 | 0.02096415 | 1 | up | yes |
| 11:10478526\|10482225 | -2.422298 | 0.02110549 | 1 | down | yes |
| 14:34783762\|34792921 | -2.4453936 | 0.0213266 | 1 | down | yes |
| 10:92180963\|92192636 | -2.5102436 | 0.02133504 | 1 | down | yes |
| 4:53383773\|53399839 | 2.11871518 | 0.02159141 | 1 | up | yes |
| 13:42963255\|42970670 | -2.7149079 | 0.02169133 | 1 | down | yes |
| 19:45022053\|45025737 | -2.336148 | 0.02173819 | 1 | down | yes |
| 20:17947486\|17957037 | 3.20707142 | 0.02183064 | 1 | up | yes |
| 2:71396141\|71418639 | -3.0206565 | 0.02232189 | 1 | down | yes |
| 10:93676653\|93687449 | -2.98961 | 0.0223867 | 1 | down | yes |
| 4:88475841\|88476030 | -2.8957542 | 0.02262354 | 1 | down | yes |
| 4:105691976\|105695668 | 2.60053265 | 0.02267481 | 1 | up | yes |
| 12:112946743\|112950975 | -2.440619 | 0.0227498 | 1 | down | yes |
| 10:119038238\|119038439 | -2.7487583 | 0.02302543 | 1 | down | yes |
| 7:84046324\|84134951 | -2.3407663 | 0.02359853 | 1 | down | yes |
| 8:17560328\|17562110 | -2.3458467 | 0.02373751 | 1 | down | yes |
| 17:54938924\|54947312 | -2.8597513 | 0.02379497 | 1 | down | yes |
| 11:74118228\|74123135 | -2.4169485 | 0.02405592 | 1 | down | yes |
| 15:68350617\|68351402 | 3.03998404 | 0.02447686 | 1 | up | yes |
| 3:9669432\|9671170 | 2.08199876 | 0.02451746 | 1 | up | yes |
| 11:46433474\|46435037 | -2.2415195 | 0.02466075 | 1 | down | yes |
| 9:128600080\|128605477 | -2.9405138 | 0.02489881 | 1 | down | yes |
| 8:105419144\|105561481 | -2.3996272 | 0.02519941 | 1 | down | yes |
| 7:80761616\|80789528 | -2.166891 | 0.02528824 | 1 | down | yes |
| 10:92111076\|92181019 | -2.5069257 | 0.02539876 | 1 | down | yes |
| 5:113101739\|113122826 | -2.2965568 | 0.02596669 | 1 | down | yes |
| 7:84129123\|84134951 | -2.6201581 | 0.02639083 | 1 | down | yes |
| 8:142330901\|142331831 | 2.07557749 | 0.02641372 | 1 | up | yes |
| 19:32643123\|32643631 | 1.90831565 | 0.0265845 | 1 | up | yes |
| 10:68032778\|68044563 | -2.4219052 | 0.02673445 | 1 | down | yes |
| X:148661908\|148662768 | -2.1368103 | 0.0269048 | 1 | down | yes |
| 2:61482380\|61490776 | 2.96362204 | 0.02691798 | 1 | up | yes |
| 14:22950314\|22952683 | 1.92384684 | 0.02762357 | 1 | up | yes |
| 3:57242856\|57267792 | 3.26020436 | 0.02873991 | 1 | up | yes |
| 5:73749837\|73753202 | -2.4817503 | 0.02895611 | 1 | down | yes |
| 6:152329730\|152331890 | 3.11537522 | 0.02926914 | 1 | up | yes |
| 12:69251129\|69262562 | 3.1726631 | 0.02929301 | 1 | up | yes |
| 17:80289659\|80290728 | -2.2898914 | 0.02972267 | 1 | down | yes |
| 3:64146830\|64163129 | -2.1584349 | 0.03002433 | 1 | down | yes |
| 1:170726220\|170730348 | 1.58844426 | 0.03152303 | 1 | up | yes |
| 4:88404876\|88405613 | -2.5143186 | 0.03157335 | 1 | down | yes |
| 5:65242104\|65273447 | -2.8364955 | 0.03181066 | 1 | down | yes |
| 1:102939035\|102946956 | -2.8923456 | 0.03182733 | 1 | down | yes |
| 10:30450258\|30451744 | -2.1542241 | 0.03222577 | 1 | down | yes |
| 11:71982761\|71990717 | -2.1780637 | 0.03320324 | 1 | down | yes |
| 13:75692417\|75727098 | 2.12444385 | 0.03337239 | 1 | up | yes |
| 2:169544721\|169557297 | 3.08890248 | 0.03405044 | 1 | up | yes |
| 1:168019545\|168023047 | 3.01222312 | 0.03408329 | 1 | up | yes |
| 14:24218133\|24218431 | 2.07394047 | 0.03422074 | 1 | up | yes |
| 2:223984752\|223992000 | -2.5224543 | 0.03433417 | 1 | down | yes |
| 3:10046580\|10049505 | 2.36151593 | 0.03444431 | 1 | up | yes |
| 13:42926336\|42970670 | -2.181505 | 0.03444618 | 1 | down | yes |
| 7:67055835\|67083539 | -2.4224598 | 0.03521641 | 1 | down | yes |
| 8:38337305\|38348215 | 2.89470144 | 0.03571296 | 1 | up | yes |
| 18:12420325\|12421629 | 2.14474309 | 0.03659215 | 1 | up | yes |
| 12:7064267\|7065299 | -2.7392927 | 0.03682162 | 1 | down | yes |
| 21:46484757\|46498833 | -2.1872198 | 0.0371715 | 1 | down | yes |
| 14:73355626\|73366981 | 2.0526393 | 0.03729233 | 1 | up | yes |
| 2:223998115\|224001922 | -2.0173228 | 0.03751089 | 1 | down | yes |
| 15:80906663\|80909306 | 1.70272444 | 0.03755474 | 1 | up | yes |
| X:109674402\|109678415 | -2.1089966 | 0.03786093 | 1 | down | yes |
| 2:160300651\|160318227 | -2.5100824 | 0.03814177 | 1 | down | yes |
| 1:32942252\|32949774 | -2.0894124 | 0.03816561 | 1 | down | yes |
| 3:52918484\|52928341 | -2.5680368 | 0.03835631 | 1 | down | yes |
| 3:195156449\|195226856 | -2.0085922 | 0.03844985 | 1 | down | yes |
| 16:53844155\|53879987 | -2.1552085 | 0.03850235 | 1 | down | yes |
| 2:223880124\|223882072 | -2.1791208 | 0.03896839 | 1 | down | yes |
| 18:6301903\|6312056 | -2.8769173 | 0.03909558 | 1 | down | yes |
| 8:22404696\|22408496 | -2.1044171 | 0.039268 | 1 | down | yes |
| 9:116235139\|116235637 | -2.3004161 | 0.03929864 | 1 | down | yes |
| 4:102304317\|102315739 | -2.1590454 | 0.03967021 | 1 | down | yes |
| 1:243695591\|243843282 | 2.17699774 | 0.03997965 | 1 | up | yes |
| 17:17214985\|17219209 | -2.533877 | 0.04022615 | 1 | down | yes |
| 17:37330173\|37330425 | 1.90239975 | 0.04027099 | 1 | up | yes |
| 1:235438769\|235442911 | 2.00810182 | 0.04080457 | 1 | up | yes |
| 12:99806404\|99825389 | -2.448017 | 0.041213 | 1 | down | yes |
| 9:5021963\|5044520 | -2.309055 | 0.04141039 | 1 | down | yes |
| 14:71618757\|71624236 | -2.0955678 | 0.0422463 | 1 | down | yes |
| 9:109263050\|109279921 | -2.7077371 | 0.04274839 | 1 | down | yes |
| 13:20051433\|20067390 | 2.86515813 | 0.0429265 | 1 | up | yes |
| 7:99386685\|99388261 | 1.95850887 | 0.04338068 | 1 | up | yes |
| 1:6599047\|6599452 | -2.0990532 | 0.04344132 | 1 | down | yes |
| 7:90716340\|90726812 | -2.1412697 | 0.04383696 | 1 | down | yes |
| 8:140347757\|140360193 | 2.76011139 | 0.04408098 | 1 | up | yes |
| 3:127694975\|127695190 | -2.7484741 | 0.04411623 | 1 | down | yes |
| 11:596923\|597570 | 1.58074963 | 0.04427124 | 1 | up | yes |
| 12:23604387\|23665564 | -2.5736014 | 0.04427432 | 1 | down | yes |
| 12:29659880\|29661379 | -2.4859603 | 0.04438713 | 1 | down | yes |
| 7:143292300\|143292698 | 1.76079796 | 0.04444695 | 1 | up | yes |
| 22:43059384\|43064324 | 1.91782781 | 0.04490208 | 1 | up | yes |
| 20:49260463\|49263483 | -2.1641233 | 0.0450386 | 1 | down | yes |
| 7:84046324\|84194668 | -2.4647375 | 0.04527876 | 1 | down | yes |
| 11:16095996\|16111926 | -2.5436476 | 0.04536368 | 1 | down | yes |
| 6:107503657\|107533610 | -2.5819749 | 0.04552637 | 1 | down | yes |
| 2:72134761\|72135419 | -2.4160492 | 0.04560267 | 1 | down | yes |
| 11:94795011\|94831541 | -2.1971266 | 0.04585686 | 1 | down | yes |
| 10:179994\|210048 | 2.84835238 | 0.04637736 | 1 | up | yes |
| 9:99959917\|99960155 | 2.73702164 | 0.0464042 | 1 | up | yes |
| 10:115461941\|115469329 | 2.26421757 | 0.0468011 | 1 | up | yes |
| 16:29904852\|29905959 | -2.104443 | 0.04687793 | 1 | down | yes |
| 18:55403454\|55464137 | -2.234238 | 0.04692819 | 1 | down | yes |
| 3:33812072\|33822082 | 2.13070195 | 0.04693107 | 1 | up | yes |
| 6:88904715\|88941180 | -2.0548488 | 0.04694821 | 1 | down | yes |
| 16:879570\|954666 | -2.158587 | 0.04698215 | 1 | down | yes |
| 8:102342473\|102361626 | 2.74391547 | 0.0470835 | 1 | up | yes |
| 15:85639374\|85645954 | -2.2731165 | 0.0474196 | 1 | down | yes |
| 2:237748364\|237760205 | -2.7026235 | 0.04752141 | 1 | down | yes |
| 5:146000058\|146014061 | -2.0209826 | 0.04813525 | 1 | down | yes |
| 10:92111076\|92192636 | -2.1164034 | 0.04818809 | 1 | down | yes |
| 12:23734684\|23741039 | -2.2073096 | 0.04824419 | 1 | down | yes |
| 14:100353687\|100361921 | -2.8844148 | 0.04870986 | 1 | down | yes |
| 9:116219937\|116227552 | -2.1449886 | 0.04880648 | 1 | down | yes |
| 13:30251931\|30255615 | 2.72122552 | 0.04895916 | 1 | up | yes |
| 1:32948222\|32949774 | -1.8993061 | 0.04897471 | 1 | down | yes |
| 5:65170617\|65188220 | -2.5143923 | 0.04906542 | 1 | down | yes |
| 19:14441143\|14441443 | 1.47758576 | 0.04925581 | 1 | up | yes |
| 9:131459164\|131459356 | 2.08756839 | 0.04941287 | 1 | up | yes |
| 10:7243558\|7285954 | -1.9542912 | 0.04979502 | 1 | down | yes |
| 7:111989015\|112004131 | -1.9814785 | 0.04990831 | 1 | down | yes |
