# Supplementary material for: Circ0083429 Regulates Osteoarthritis Progression via the Mir-346/SMAD3 Axis
Source: Front Cell Dev Biol. 2021 Jan 15;8:579945. doi: 10.3389/fcell.2020.579945 (PMC7843588; doi:10.3389/fcell.2020.579945)
Supplement: Supplementary file 2 [file Table_2.docx]

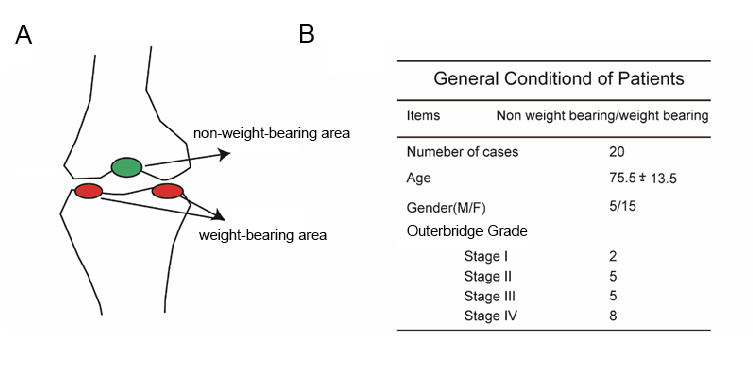


Supplement Figure 1

1. Model diagram of chondrocyte selection location. The blue is non bearing area, act as normal cartilage(control). The red is bearing area, act as osteoarthritis (OA).
2. Basic information and Outerbrige Grade scores of 20 patients.


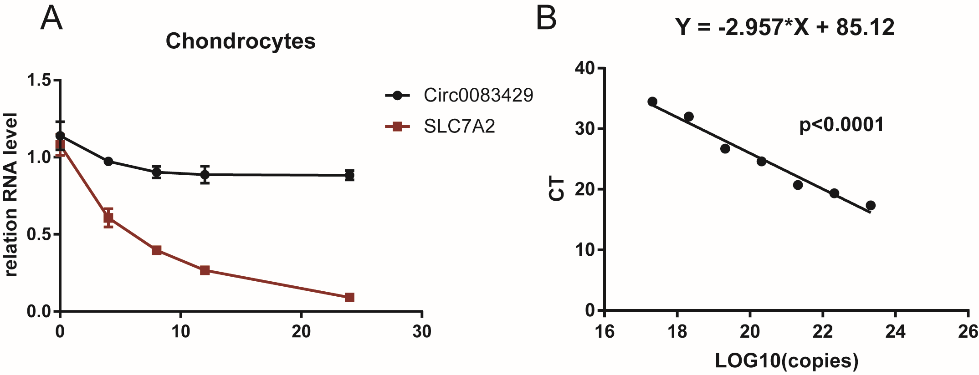


Supplement Figure 2

1. Actinomycin D assay to measure the stability of RNA at different time of 4h, 8h, 12h and 24h. The host gene SLC7A2 of circ0083429 was used as a positive control for linear RNA.
2. The standard curve described the relationship between CT value and LOG (copies) of Circ0083429 in chondrocytes.


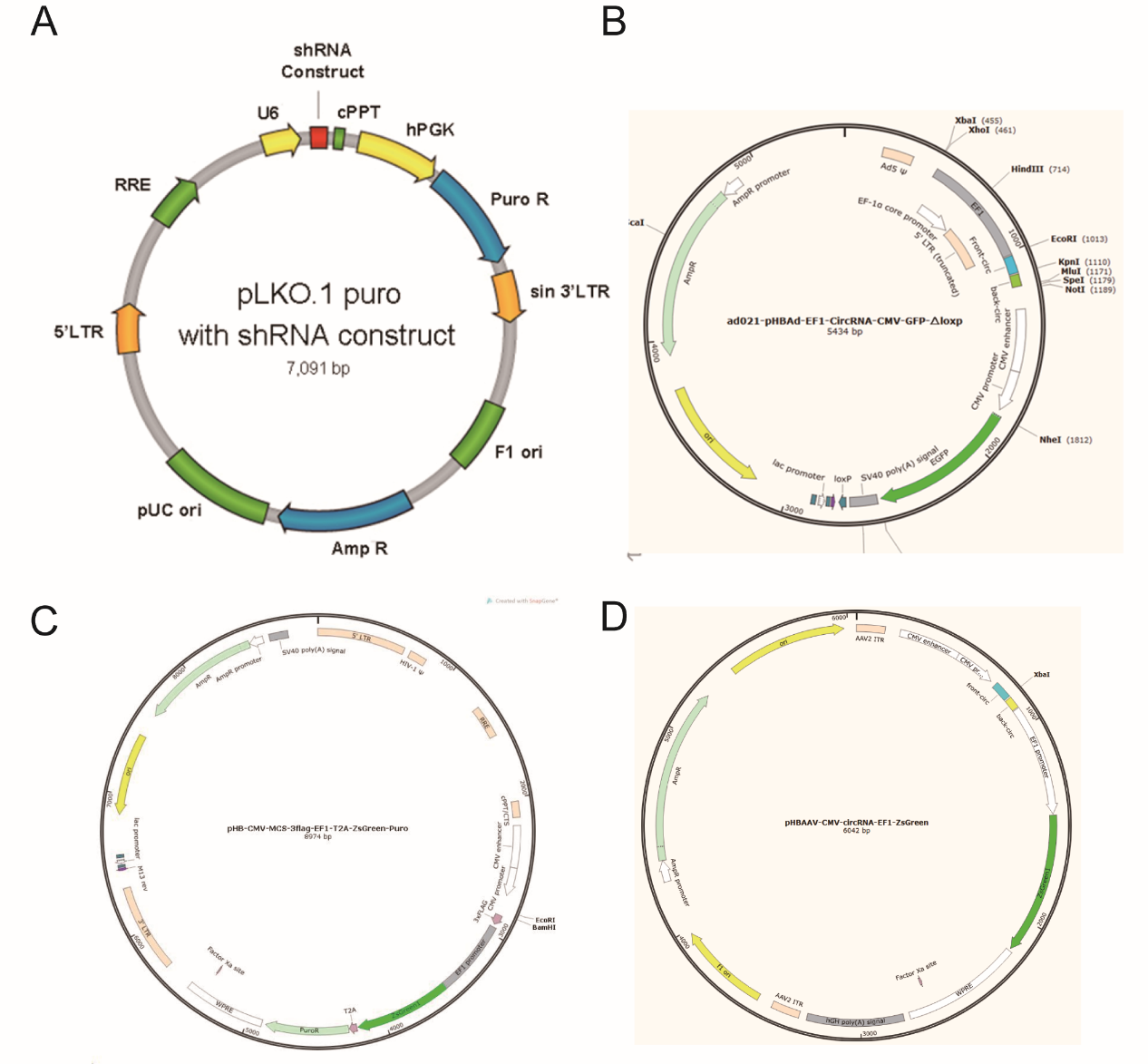


Supplement Figure 3

A The structure of PLKO.1 for ShRNA construction.

B The structure of plasmid for Circ0083429 overexpression.

C The structure of plasmid for *SMAD3* overexpression.

D Adenovirus plasmid structure overexpressing Circ0083429.

A


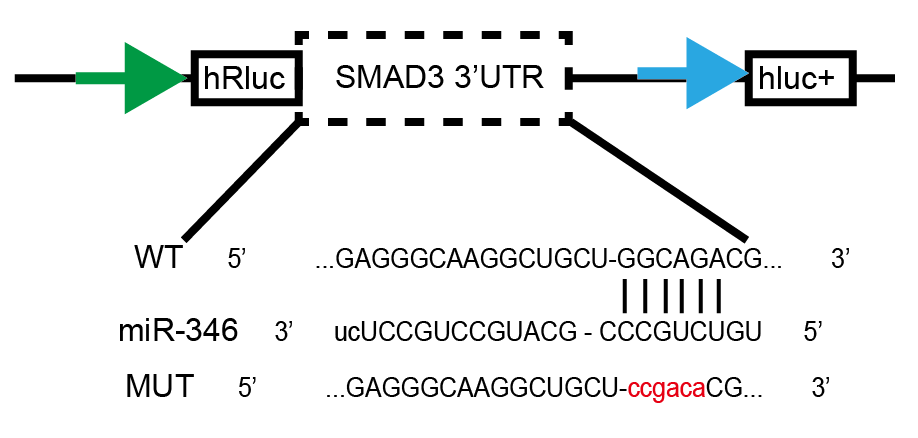


Supplement Figure 4

A Luciferase reporter assay was used to evaluate the luciferase activity of LUC-SMAD3 WT and LUC-SMAD3 mutant (MUT) in HEK-293T cells co-transfected with miR-346 mimic or mimic NC. Data represent mean ± SD (n=3); ^*^*P < 0.05*.
