# Supplementary material for: Circ0083429 Regulates Osteoarthritis Progression via the Mir-346/SMAD3 Axis
Source: Front Cell Dev Biol. 2021 Jan 15;8:579945. doi: 10.3389/fcell.2020.579945 (PMC7843588; doi:10.3389/fcell.2020.579945)
Supplement: Supplementary file 3 [file Data_Sheet_1.PDF]

Generated at: 2019/12/18 02:35:11
mirDIP version: 4.1.11.1.
Database version: 4.1.0.3

Gene Symbol -
Micro RNA - hsa-miR-346
Minimum Score - Very High

Results (382)

Gene Sym	Uniprot	Pseudoge	MicroRNA	Integrated	Number o	Score Clas	Sources
ZFC3H1	O60293		hsa-miR-3	0.807648	19	Very High	BCmicrO|BiTargeting
CSNK1D	P48730		hsa-miR-3	0.796594	16	Very High	BCmicrO|BiTargeting
BCL6	P41182		hsa-miR-3	0.796529	18	Very High	BCmicrO|CoMeTa|DI/
RDX	P35241		hsa-miR-3	0.758307	20	Very High	BCmicrO|BiTargeting
LRP6	O75581		hsa-miR-3	0.753726	21	Very High	BCmicrO|BiTargeting
OSER1	Q9NX31		hsa-miR-3	0.732855	16	Very High	BCmicrO|CoMeTa|DI/
RIMS2	Q9UQ26		hsa-miR-3	0.723068	17	Very High	BCmicrO|BiTargeting
SZRD1	Q7Z422		hsa-miR-3	0.698874	17	Very High	BCmicrO|BiTargeting
NFIB	O00712		hsa-miR-3	0.698861	18	Very High	BCmicrO|BiTargeting
LHX6	Q9UPM6		hsa-miR-3	0.696687	17	Very High	BCmicrO|CoMeTa|DI/
AGO2	Q9UKV8		hsa-miR-3	0.694112	20	Very High	BCmicrO|BiTargeting
CPSF7	Q8N684		hsa-miR-3	0.691768	16	Very High	BCmicrO|BiTargeting
CAND1	Q86VP6		hsa-miR-3	0.685614	18	Very High	BCmicrO|BiTargeting
SORCS3	Q9UPU3		hsa-miR-3	0.683834	16	Very High	BCmicrO|BiTargeting
NXPH1	P58417		hsa-miR-3	0.675371	17	Very High	BCmicrO|BiTargeting
PHC1	P78364		hsa-miR-3	0.673299	14	Very High	BCmicrO|BiTargeting
HBP1	O60381		hsa-miR-3	0.670525	16	Very High	BCmicrO|CoMeTa|Cu
NCOA7	Q8NI08		hsa-miR-3	0.667956	17	Very High	BCmicrO|BiTargeting
MBNL1	Q9NR56		hsa-miR-3	0.666933	15	Very High	BCmicrO|CoMeTa|DI/
FAM131A	Q6UXB0		hsa-miR-3	0.664502	17	Very High	BCmicrO|BiTargeting
SLC38A4	Q969I6		hsa-miR-3	0.6589	17	Very High	BCmicrO|BiTargeting
GID8	Q9NWU2		hsa-miR-3	0.654598	15	Very High	BCmicrO|CoMeTa|DI/
KCNC4	Q03721		hsa-miR-3	0.650428	17	Very High	BCmicrO|BiTargeting
MEMO1	Q9Y316		hsa-miR-3	0.650306	15	Very High	BCmicrO|CoMeTa|DI/
FGF7	P21781		hsa-miR-3	0.649855	15	Very High	BCmicrO|BiTargeting
IER5	Q5VY09		hsa-miR-3	0.644788	17	Very High	BCmicrO|BiTargeting
PTPN5	P54829		hsa-miR-3	0.639832	15	Very High	BCmicrO|BiTargeting
HOXA7	P31268		hsa-miR-3	0.638676	13	Very High	BCmicrO|Cupid|DIAN
SRSF10	O75494		hsa-miR-3	0.636348	16	Very High	BCmicrO|CoMeTa|Cu
SFRP4	Q6FHJ7		hsa-miR-3	0.634252	13	Very High	BCmicrO|CoMeTa|DI/
ZDHHC13	Q8IUH4		hsa-miR-3	0.632263	13	Very High	BCmicrO|DIANA|EIMI
UTP6	Q9NYH9		hsa-miR-3	0.625932	15	Very High	BCmicrO|DIANA|EIMI
ZNF664	Q8N3J9		hsa-miR-3	0.618524	13	Very High	BCmicrO|DIANA|EIMI
NIN	Q8N4C6		hsa-miR-3	0.615818	17	Very High	BCmicrO|CoMeTa|Cu
SF1	Q15637		hsa-miR-3	0.614793	13	Very High	BCmicrO|CoMeTa|DI/
LIF	P15018		hsa-miR-3	0.609554	12	Very High	BCmicrO|BiTargeting
SMAD3	P84022		hsa-miR-3	0.608897	18	Very High	BCmicrO|BiTargeting
CCNL1	Q9UK58		hsa-miR-3	0.60874	14	Very High	BCmicrO|CoMeTa|Cu
SLC6A6	P31641		hsa-miR-3	0.604754	15	Very High	BCmicrO|BiTargeting
PAK1IP1	Q9NWT1		hsa-miR-3	0.602982	14	Very High	BCmicrO|Cupid|DIAN
ENTPD1	P49961		hsa-miR-3	0.60252	14	Very High	BCmicrO|CoMeTa|DI/
CADM3	Q8N126		hsa-miR-3	0.598839	14	Very High	BCmicrO|CoMeTa|DI/
ACVR2B	Q13705		hsa-miR-3	0.597087	14	Very High	BCmicrO|BiTargeting
FAM53B	Q14153		hsa-miR-3	0.595938	17	Very High	BCmicrO|BiTargeting
KIF1B	O60333		hsa-miR-3	0.592762	17	Very High	BCmicrO|BiTargeting

RAPGEF1	Q13905	hsa-miR-3	0.590568	13	Very High	BCmicrO|BiTargeting
NCDN	Q9UBB6	hsa-miR-3	0.588277	16	Very High	BCmicrO|BiTargeting
ARF4	P18085	hsa-miR-3	0.583053	14	Very High	BCmicrO|CoMeTa|DIA
KIF5C	O60282	hsa-miR-3	0.582262	16	Very High	BCmicrO|BiTargeting
KCNA3	P22001	hsa-miR-3	0.581371	13	Very High	BCmicrO|CoMeTa|DIA
EPB41L1	Q9H4G0	hsa-miR-3	0.577653	16	Very High	BCmicrO|BiTargeting
PRICKLE2	Q7Z3G6	hsa-miR-3	0.576345	16	Very High	BCmicrO|BiTargeting
NMT1	P30419	hsa-miR-3	0.573433	14	Very High	BCmicrO|CoMeTa|DIA
SPRYD7	Q5W111	hsa-miR-3	0.571191	13	Very High	BCmicrO|CoMeTa|DIA
NFAM1	Q8NET5	hsa-miR-3	0.570857	14	Very High	BCmicrO|BiTargeting
SMYD3	Q9H7B4	hsa-miR-3	0.57012	16	Very High	BCmicrO|BiTargeting
BMP8B	P34820	hsa-miR-3	0.56807	14	Very High	BCmicrO|BiTargeting
PTCHD1	Q96NR3	hsa-miR-3	0.565969	18	Very High	BCmicrO|BiTargeting
POU2F1	P14859	hsa-miR-3	0.564649	16	Very High	BCmicrO|BiTargeting
PGK1	P00558	hsa-miR-3	0.564229	13	Very High	BCmicrO|BiTargeting
AP5M1	Q9H0R1	hsa-miR-3	0.563686	13	Very High	BCmicrO|DIANA|EIMI
GOLGA7B	Q2TAP0	hsa-miR-3	0.560862	12	Very High	BCmicrO|DIANA|EIMI
ZNF609	O15014	hsa-miR-3	0.56036	14	Very High	BCmicrO|BiTargeting
PDZD4	Q76G19	hsa-miR-3	0.558036	15	Very High	BCmicrO|BiTargeting
PRRC2B	Q5JSZ5	hsa-miR-3	0.557499	15	Very High	BCmicrO|BiTargeting
MAN1C1	Q9NR34	hsa-miR-3	0.556989	16	Very High	BCmicrO|BiTargeting
CALN1	Q9BXU9	hsa-miR-3	0.556234	15	Very High	BCmicrO|BiTargeting
OAS2	P29728	hsa-miR-3	0.55503	15	Very High	BCmicrO|BiTargeting
EIF3J	O75822	hsa-miR-3	0.554782	13	Very High	BCmicrO|CoMeTa|DIA
PTPN18	Q99952	hsa-miR-3	0.552789	13	Very High	BCmicrO|DIANA|EIMI
IL20RA	Q9UHF4	hsa-miR-3	0.552462	14	Very High	BCmicrO|CoMeTa|DIA
SHANK2	Q9UPX8	hsa-miR-3	0.552055	15	Very High	BCmicrO|BiTargeting
NLGN2	Q8NFZ4	hsa-miR-3	0.551723	15	Very High	BCmicrO|BiTargeting
MMD2	Q8IY49	hsa-miR-3	0.550478	14	Very High	BCmicrO|BiTargeting
KMT2D	O14686	hsa-miR-3	0.548241	13	Very High	BCmicrO|CoMeTa|DIA
ZC3H12C	Q9C0D7	hsa-miR-3	0.54816	14	Very High	BCmicrO|DIANA|EIMI
PPP1R16B	Q96T49	hsa-miR-3	0.546703	13	Very High	BCmicrO|CoMeTa|DIA
ZNF148	Q9UQR1	hsa-miR-3	0.546076	15	Very High	BCmicrO|DIANA|EIMI
LTBP1	Q14766	hsa-miR-3	0.545225	15	Very High	BCmicrO|CoMeTa|DIA
RPRD1A	Q96P16	hsa-miR-3	0.543665	15	Very High	BCmicrO|CoMeTa|DIA
TRAF4	Q9BUZ4	hsa-miR-3	0.538325	15	Very High	BCmicrO|BiTargeting
MASP1	P48740	hsa-miR-3	0.53797	16	Very High	BCmicrO|BiTargeting
CREB3L3	Q68CJ9	hsa-miR-3	0.537134	14	Very High	BCmicrO|BiTargeting
PAFAH2	Q99487	hsa-miR-3	0.536564	15	Very High	BCmicrO|BiTargeting
GSK3B	P49841	hsa-miR-3	0.53557	13	Very High	BCmicrO|BiTargeting
PKIA	P61925	hsa-miR-3	0.534862	16	Very High	BCmicrO|BiTargeting
PRKAB2	O43741	hsa-miR-3	0.534487	17	Very High	BCmicrO|BiTargeting
CCSAP	Q6IQ19	hsa-miR-3	0.533019	13	Very High	BCmicrO|BiTargeting
YTHDF1	Q9BYJ9	hsa-miR-3	0.527526	12	Very High	BCmicrO|DIANA|EIMI
CYB561D1	Q8N8Q1	hsa-miR-3	0.527361	16	Very High	BCmicrO|BiTargeting
REPS2	Q8NFH8	hsa-miR-3	0.527024	15	Very High	BCmicrO|CoMeTa|DIA
FRAS1	Q86XX4	hsa-miR-3	0.5254	16	Very High	BCmicrO|BiTargeting
LIN54	Q6MZP7	hsa-miR-3	0.523317	13	Very High	BCmicrO|BiTargeting
SNX10	Q9Y5X0	hsa-miR-3	0.52109	13	Very High	BCmicrO|CoMeTa|DIA
CABP7	Q86V35	hsa-miR-3	0.518502	15	Very High	BCmicrO|BiTargeting
SLC25A36	Q96CQ1	hsa-miR-3	0.518031	16	Very High	BCmicrO|BiTargeting
EGLN3	Q9H6Z9	hsa-miR-3	0.517876	16	Very High	BCmicrO|BiTargeting
MKL2	Q9ULH7	hsa-miR-3	0.517098	12	Very High	BCmicrO|DIANA|EIMI
FAM155B	O75949	hsa-miR-3	0.515897	14	Very High	BCmicrO|BiTargeting
ADAMTS6	Q9UKP5	hsa-miR-3	0.515069	14	Very High	BCmicrO|BiTargeting
MMP24	Q9Y5R2	hsa-miR-3	0.514676	14	Very High	BCmicrO|BiTargeting
RCN1	Q15293	hsa-miR-3	0.512696	13	Very High	BCmicrO|CoMeTa|DIA
SAMD12	Q8N8I0	hsa-miR-3	0.512679	13	Very High	BCmicrO|DIANA|EIMI

ANKS6	Q68DC2	hsa-miR-3	0.511114	14	Very High	BCmicrO|BiTargeting
NMB	P08949	hsa-miR-3	0.510602	14	Very High	BCmicrO|CoMeTa|DI/
CPEB4	Q17RY0	hsa-miR-3	0.51028	14	Very High	BCmicrO|Cupid|DIAN
FGFRL1	Q8N441	hsa-miR-3	0.509881	15	Very High	BCmicrO|BiTargeting
ARF1	P84077	hsa-miR-3	0.508838	16	Very High	BCmicrO|BiTargeting
ATP2A2	P16615	hsa-miR-3	0.503849	14	Very High	BCmicrO|Cupid|DIAN
REXO4	Q9GZR2	hsa-miR-3	0.5017	15	Very High	BCmicrO|BiTargeting
LIMD2	Q9BT23	hsa-miR-3	0.500994	12	Very High	BCmicrO|CoMeTa|DI/
COG3	Q96JB2	hsa-miR-3	0.500328	14	Very High	BCmicrO|BiTargeting
RFLNB	Q8N5W9	hsa-miR-3	0.500152	13	Very High	BCmicrO|DIANA|EIMI
SETD7	Q8WTS6	hsa-miR-3	0.499849	15	Very High	BCmicrO|BiTargeting
BORCS5	Q969J3	hsa-miR-3	0.498966	14	Very High	BCmicrO|BiTargeting
OXNAD1	Q96HP4	hsa-miR-3	0.496195	17	Very High	BCmicrO|BiTargeting
SASH1	O94885	hsa-miR-3	0.495323	13	Very High	BCmicrO|CoMeTa|DI/
MEF2A	Q02078	hsa-miR-3	0.494985	13	Very High	BCmicrO|BiTargeting
KIAA1328	Q86T90	hsa-miR-3	0.494399	14	Very High	BCmicrO|DIANA|EIMI
RBM39	Q14498	hsa-miR-3	0.492748	14	Very High	BCmicrO|BiTargeting
IBA57	Q5T440	hsa-miR-3	0.491392	13	Very High	BCmicrO|BiTargeting
KLF7	O75840	hsa-miR-3	0.491325	12	Very High	BCmicrO|CoMeTa|DI/
RNF125	Q96EQ8	hsa-miR-3	0.490997	14	Very High	BCmicrO|CoMeTa|DI/
ETV6	P41212	hsa-miR-3	0.49087	12	Very High	BCmicrO|CoMeTa|EIN
BAZ2B	Q9UIF8	hsa-miR-3	0.489385	12	Very High	BCmicrO|Cupid|DIAN
ANKRD12	Q6UB98	hsa-miR-3	0.488702	16	Very High	BCmicrO|BiTargeting
BRWD1	Q9NSI6	hsa-miR-3	0.486639	14	Very High	BCmicrO|CoMeTa|DI/
NAALAD2	Q9Y3Q0	hsa-miR-3	0.486193	11	Very High	BCmicrO|DIANA|EIMI
AP3S2	P59780	hsa-miR-3	0.486068	14	Very High	BCmicrO|BiTargeting
FNTB	P49356	hsa-miR-3	0.48522	13	Very High	BCmicrO|BiTargeting
MRPS35	P82673	hsa-miR-3	0.482365	10	Very High	BCmicrO|Cupid|DIAN
DLG2	Q15700	hsa-miR-3	0.480884	13	Very High	BCmicrO|CoMeTa|DI/
PBRM1	Q86U86	hsa-miR-3	0.480703	14	Very High	BCmicrO|BiTargeting
PROK1	P58294	hsa-miR-3	0.480131	15	Very High	BCmicrO|DIANA|EIMI
PITPNA	Q00169	hsa-miR-3	0.479992	12	Very High	BCmicrO|CoMeTa|DI/
RASSF1	Q9NS23	hsa-miR-3	0.477714	15	Very High	BCmicrO|BiTargeting
PDPK1	O15530	hsa-miR-3	0.477703	15	Very High	BCmicrO|BiTargeting
KCTD15	Q96SI1	hsa-miR-3	0.477281	13	Very High	BCmicrO|CoMeTa|DI/
KLHL3	Q9UH77	hsa-miR-3	0.476361	12	Very High	BCmicrO|CoMeTa|DI/
HES7	Q9BYE0	hsa-miR-3	0.475744	14	Very High	BCmicrO|BiTargeting
PSG3	Q16557	hsa-miR-3	0.473859	14	Very High	BCmicrO|CoMeTa|DI/
ATP8B4	Q8TF62	hsa-miR-3	0.472881	13	Very High	BCmicrO|BiTargeting
MRPS9	P82933	hsa-miR-3	0.472004	13	Very High	BCmicrO|EIMMo3|M/
ERLEC1	Q96DZ1	hsa-miR-3	0.471477	12	Very High	BCmicrO|DIANA|EIMI
GRM7	Q14831	hsa-miR-3	0.471305	12	Very High	BCmicrO|CoMeTa|DI/
MTPN	P58546	hsa-miR-3	0.47105	12	Very High	BCmicrO|DIANA|EIMI
MFAP5	Q13361	hsa-miR-3	0.469915	11	Very High	BCmicrO|CoMeTa|DI/
RET	P07949	hsa-miR-3	0.469831	14	Very High	BCmicrO|CoMeTa|DI/
CEACAM8	P31997	hsa-miR-3	0.468488	12	Very High	BCmicrO|CoMeTa|DI/
CIB2	O75838	hsa-miR-3	0.468112	14	Very High	BCmicrO|DIANA|EIMI
PFN2	P35080	hsa-miR-3	0.467951	12	Very High	BCmicrO|BiTargeting
SPIN3	Q5JUX0	hsa-miR-3	0.467914	13	Very High	BCmicrO|BiTargeting
EPAS1	Q99814	hsa-miR-3	0.467314	13	Very High	BCmicrO|CoMeTa|DI/
ROCK1	Q13464	hsa-miR-3	0.466991	11	Very High	BCmicrO|Cupid|DIAN
AJAP1	Q9UKB5	hsa-miR-3	0.466986	13	Very High	BCmicrO|BiTargeting
URM1	Q9BTM9	hsa-miR-3	0.466363	14	Very High	BCmicrO|BiTargeting
ADAM12	O43184	hsa-miR-3	0.465519	14	Very High	BCmicrO|BiTargeting
BNC2	Q6ZN30	hsa-miR-3	0.465326	12	Very High	BCmicrO|BiTargeting
ZNF382	Q96SR6	hsa-miR-3	0.465054	12	Very High	BCmicrO|DIANA|EIMI
NUDT13	Q86X67	hsa-miR-3	0.464395	9	Very High	BCmicrO|CoMeTa|DI/
ITPKB	P27987	hsa-miR-3	0.462656	15	Very High	BCmicrO|BiTargeting

PRRX2	Q99811	hsa-miR-3	0.462083	10	Very	High	BCmicrO|ElMMo3|MA
LPIN1	Q14693	hsa-miR-3	0.461839	13	Very	High	BCmicrO|DIANA|ElMI
SLAMF1	Q13291	hsa-miR-3	0.461665	12	Very	High	BCmicrO|CoMeTa|DIA
TULP4	Q9NRJ4	hsa-miR-3	0.461254	14	Very	High	BCmicrO|BiTargeting
DKK3	Q9UBP4	hsa-miR-3	0.460022	13	Very	High	BCmicrO|CoMeTa|DIA
ZIC1	Q15915	hsa-miR-3	0.45972	13	Very	High	BCmicrO|CoMeTa|DIA
PHLPP2	Q6ZVD8	hsa-miR-3	0.459514	14	Very	High	BCmicrO|CoMeTa|DIA
CNOT2	Q9NZN8	hsa-miR-3	0.45937	12	Very	High	BCmicrO|CoMeTa|Cu
C14orf177	Q52M58	hsa-miR-3	0.459259	11	Very	High	BCmicrO|DIANA|ElMI
SPINK2	P20155	hsa-miR-3	0.459038	13	Very	High	BCmicrO|CoMeTa|DIA
DLST	P36957	hsa-miR-3	0.457999	13	Very	High	BCmicrO|BiTargeting
MAP7D1	Q3KQU3	hsa-miR-3	0.457385	15	Very	High	BCmicrO|BiTargeting
GTPBP1	O00178	hsa-miR-3	0.457221	13	Very	High	BCmicrO|BiTargeting
PPP1R9B	Q96SB3	hsa-miR-3	0.457183	10	Very	High	BCmicrO|ElMMo3|ME
UBE2Q2	Q8WVN8	hsa-miR-3	0.456836	12	Very	High	BCmicrO|Cupid|DIAN
THUMPD1	Q9NXG2	hsa-miR-3	0.456741	13	Very	High	BCmicrO|DIANA|ElMI
EXTL1	Q92935	hsa-miR-3	0.456436	13	Very	High	BCmicrO|DIANA|ElMI
HNRNPA1	P09651	hsa-miR-3	0.456356	9	Very	High	BCmicrO|BiTargeting
ADAMTS1	Q8TE56	hsa-miR-3	0.45632	15	Very	High	BCmicrO|BiTargeting
PVR	P15151	hsa-miR-3	0.455801	15	Very	High	BCmicrO|BiTargeting
IRAK1	P51617	hsa-miR-3	0.455049	13	Very	High	BCmicrO|BiTargeting
PKNOX2	Q96KN3	hsa-miR-3	0.454817	13	Very	High	BCmicrO|BiTargeting
INTS2	Q9H0H0	hsa-miR-3	0.454583	12	Very	High	BCmicrO|DIANA|ElMI
SYNM	O15061	hsa-miR-3	0.454202	13	Very	High	BCmicrO|DIANA|ElMI
NIPAL3	Q6P499	hsa-miR-3	0.453883	14	Very	High	BCmicrO|DIANA|ElMI
ZBTB8B	Q8NAP8	hsa-miR-3	0.453615	13	Very	High	BiTargeting|DIANA|El
SPRY3	O43610	hsa-miR-3	0.452158	13	Very	High	BCmicrO|BiTargeting
ASPHD2	Q6ICH7	hsa-miR-3	0.451791	13	Very	High	BCmicrO|BiTargeting
FAM53C	Q9NYF3	hsa-miR-3	0.451668	12	Very	High	BCmicrO|CoMeTa|DIA
TXNRD1	Q16881	hsa-miR-3	0.451409	13	Very	High	BCmicrO|DIANA|ElMI
SENP5	Q96HI0	hsa-miR-3	0.45132	14	Very	High	BCmicrO|BiTargeting
MYEF2	Q9P2K5	hsa-miR-3	0.451064	16	Very	High	BCmicrO|BiTargeting
FLNB	O75369	hsa-miR-3	0.450954	15	Very	High	BCmicrO|BiTargeting
KLHL1	Q9NR64	hsa-miR-3	0.450067	13	Very	High	BCmicrO|CoMeTa|DIA
DBNL	Q9UJU6	hsa-miR-3	0.450022	12	Very	High	BCmicrO|BiTargeting
TMEM9B	Q9NQ34	hsa-miR-3	0.449303	12	Very	High	BCmicrO|DIANA|ElMI
MAPK14	Q16539	hsa-miR-3	0.449016	12	Very	High	BCmicrO|BiTargeting
NDST1	P52848	hsa-miR-3	0.448221	12	Very	High	BCmicrO|DIANA|ElMI
IGFBP5	P24593	hsa-miR-3	0.447928	13	Very	High	BCmicrO|BiTargeting
DCTN4	Q9UJW0	hsa-miR-3	0.447568	13	Very	High	BCmicrO|BiTargeting
PSMG3	Q9BT73	hsa-miR-3	0.44749	14	Very	High	BCmicrO|DIANA|ElMI
ATP1A2	P50993	hsa-miR-3	0.445308	14	Very	High	BCmicrO|BiTargeting
ICA1L	Q8NDH6	hsa-miR-3	0.445289	14	Very	High	BCmicrO|BiTargeting
CA7	P43166	hsa-miR-3	0.444311	12	Very	High	BCmicrO|BiTargeting
SCML4	Q8N228	hsa-miR-3	0.443632	13	Very	High	BCmicrO|DIANA|ElMI
SPDYE3	A6NKU9	hsa-miR-3	0.443562	11	Very	High	BCmicrO|DIANA|ElMI
IL13	P35225	hsa-miR-3	0.443143	14	Very	High	BCmicrO|BiTargeting
F7	P08709	hsa-miR-3	0.441699	14	Very	High	BCmicrO|BiTargeting
SERPINH1	P50454	hsa-miR-3	0.441212	13	Very	High	BCmicrO|BiTargeting
KCTD5	Q9NXV2	hsa-miR-3	0.44114	14	Very	High	BCmicrO|DIANA|ElMI
PLSCR4	Q9NRQ2	hsa-miR-3	0.4407	13	Very	High	BCmicrO|DIANA|ElMI
POLB	P06746	hsa-miR-3	0.439527	12	Very	High	BCmicrO|BiTargeting
RGS4	P49798	hsa-miR-3	0.438338	15	Very	High	BCmicrO|BiTargeting
KCNH6	Q9H252	hsa-miR-3	0.437558	10	Very	High	BCmicrO|BiTargeting
SH3PXD2A	Q5TCZ1	hsa-miR-3	0.437169	15	Very	High	BCmicrO|BiTargeting
KCTD12	Q96CX2	hsa-miR-3	0.437134	11	Very	High	BCmicrO|DIANA|ElMI
ANKRD52	Q8NB46	hsa-miR-3	0.437006	11	Very	High	BCmicrO|BiTargeting
PSG5	Q15238	hsa-miR-3	0.435964	13	Very	High	BCmicrO|CoMeTa|ElM

ABCC12	Q96J65	hsa-miR-3	0.435846	11	Very High	BCmicrO|BiTargeting
MAP4K4	O95819	hsa-miR-3	0.435639	12	Very High	BCmicrO|BiTargeting
MRPL11	Q9Y3B7	hsa-miR-3	0.435366	13	Very High	BCmicrO|BiTargeting
PEG10	Q86TG7	hsa-miR-3	0.434753	12	Very High	BCmicrO|BiTargeting
CARHSP1	Q9Y2V2	hsa-miR-3	0.43384	16	Very High	BCmicrO|BiTargeting
WWC2	Q6AWC2	hsa-miR-3	0.433437	13	Very High	BCmicrO|DIANA|EIMI
UBE2G2	P60604	hsa-miR-3	0.432692	14	Very High	BCmicrO|DIANA|EIMI
DNAJB3	Q8WWF6	hsa-miR-3	0.432444	9	Very High	BCmicrO|EIMMo3|mi
KLF15	Q9UIH9	hsa-miR-3	0.432013	13	Very High	BCmicrO|DIANA|EIMI
STX1B	P61266	hsa-miR-3	0.43201	12	Very High	BCmicrO|CoMeTa|DI
CRTC1	Q6UUV9	hsa-miR-3	0.430681	14	Very High	BCmicrO|BiTargeting
CDKN2B	P42772	hsa-miR-3	0.429689	14	Very High	BCmicrO|BiTargeting
RNF165	Q6ZSG1	hsa-miR-3	0.429548	11	Very High	BCmicrO|BiTargeting
KLF4	O43474	hsa-miR-3	0.428987	11	Very High	BCmicrO|CoMeTa|DI
HRASLS	Q9HDD0	hsa-miR-3	0.428936	11	Very High	BCmicrO|CoMeTa|EIN
MTERF4	Q7Z6M4	hsa-miR-3	0.428358	11	Very High	BCmicrO|DIANA|EIMI
USP40	Q9NVE5	hsa-miR-3	0.428114	14	Very High	BCmicrO|BiTargeting
ARHGEF15	O94989	hsa-miR-3	0.427988	12	Very High	BCmicrO|BiTargeting
DAND5	Q8N907	hsa-miR-3	0.427497	13	Very High	BCmicrO|BiTargeting
FRMD4B	Q9Y2L6	hsa-miR-3	0.427193	13	Very High	BCmicrO|CoMeTa|DI
EGLN2	Q96KS0	hsa-miR-3	0.427062	12	Very High	BCmicrO|CoMeTa|DI
NRP1	O14786	hsa-miR-3	0.427035	12	Very High	BCmicrO|BiTargeting
C1orf122	Q6ZSJ8	hsa-miR-3	0.426937	13	Very High	BCmicrO|DIANA|EIMI
CBX7	O95931	hsa-miR-3	0.426759	15	Very High	BCmicrO|BiTargeting
ST6GAL1	P15907	hsa-miR-3	0.426383	13	Very High	BCmicrO|CoMeTa|DI
KRAS	P01116	hsa-miR-3	0.425921	13	Very High	BCmicrO|CoMeTa|DI
PGAP1	Q75T13	hsa-miR-3	0.425283	14	Very High	BCmicrO|BiTargeting
CSK	P41240	hsa-miR-3	0.424671	14	Very High	BCmicrO|BiTargeting
ZNF177	Q13360	hsa-miR-3	0.424586	13	Very High	BCmicrO|DIANA|EIMI
NPHS2	Q9NP85	hsa-miR-3	0.424137	13	Very High	BCmicrO|DIANA|EIMI
CLEC4F	Q8N1N0	hsa-miR-3	0.423986	14	Very High	BCmicrO|BiTargeting
ITCH	Q96J02	hsa-miR-3	0.423924	12	Very High	BCmicrO|DIANA|EIMI
4-Sep	O43236	hsa-miR-3	0.423819	12	Very High	BCmicrO|CoMeTa|DI
TNFRSF12	Q9NP84	hsa-miR-3	0.422871	12	Very High	BCmicrO|CoMeTa|DI
CCDC144I	Q6NUI1	hsa-miR-3	0.421742	11	Very High	BCmicrO|DIANA|EIMI
MYO5A	Q9Y4I1	hsa-miR-3	0.421206	12	Very High	BCmicrO|BiTargeting
TTC9	Q92623	hsa-miR-3	0.420424	11	Very High	BCmicrO|CoMeTa|DI
GCFC2	P16383	hsa-miR-3	0.419992	12	Very High	BCmicrO|DIANA|EIMI
SCNN1A	P37088	hsa-miR-3	0.41903	13	Very High	BCmicrO|BiTargeting
IP6K3	Q96PC2	hsa-miR-3	0.418897	12	Very High	BCmicrO|BiTargeting
CCDC136	Q96JN2	hsa-miR-3	0.418552	11	Very High	BCmicrO|DIANA|EIMI
COL2A1	P02458	hsa-miR-3	0.418495	11	Very High	BCmicrO|CoMeTa|DI
TOMM40L	Q969M1	hsa-miR-3	0.418119	15	Very High	BCmicrO|BiTargeting
ZNF248	Q8NDW4	hsa-miR-3	0.41787	13	Very High	BCmicrO|BiTargeting
SPOUT1	Q5T280	hsa-miR-3	0.41761	12	Very High	BCmicrO|BiTargeting
KCTD6	Q8NC69	hsa-miR-3	0.417254	14	Very High	BCmicrO|BiTargeting
SGTA	O43765	hsa-miR-3	0.417104	11	Very High	BCmicrO|DIANA|EIMI
ELFN2	Q5R3F8	hsa-miR-3	0.415426	12	Very High	BCmicrO|BiTargeting
MOCS2	O96007	hsa-miR-3	0.414347	12	Very High	BCmicrO|BiTargeting
DYRK1A	Q13627	hsa-miR-3	0.413948	12	Very High	BCmicrO|DIANA|EIMI
PCBP4	P57723	hsa-miR-3	0.413633	14	Very High	BCmicrO|BiTargeting
SLC9A3R1	O14745	hsa-miR-3	0.413357	11	Very High	BCmicrO|CoMeTa|DI
PCTP	Q9UKL6	hsa-miR-3	0.413335	11	Very High	BCmicrO|CoMeTa|DI
CARM1	Q86X55	hsa-miR-3	0.413108	14	Very High	BCmicrO|BiTargeting
C6orf106	Q9H6K1	hsa-miR-3	0.41308	13	Very High	BCmicrO|BiTargeting
FBXL2	Q9UKC9	hsa-miR-3	0.412943	10	Very High	BCmicrO|DIANA|EIMI
SETD4	Q9NVD3	hsa-miR-3	0.412115	13	Very High	BCmicrO|BiTargeting
PPP4R1	Q8TF05	hsa-miR-3	0.411332	13	Very High	BCmicrO|CoMeTa|DI

ONECUT2	O95948	hsa-miR-3	0.411079	12	Very High	BCmicrO|BiTargeting
ROR1	Q01973	hsa-miR-3	0.410941	12	Very High	BCmicrO|Cupid|DIAN
ABCG4	Q9H172	hsa-miR-3	0.410797	11	Very High	BCmicrO|CoMeTa|DI
SPICE1	Q8N0Z3	hsa-miR-3	0.410751	12	Very High	BCmicrO|DIANA|ElMI
ELF2	Q15723	hsa-miR-3	0.409997	11	Very High	BCmicrO|Cupid|DIAN
IGFL3	Q6UXB1	hsa-miR-3	0.40994	12	Very High	BCmicrO|DIANA|ElMI
LARP4B	Q92615	hsa-miR-3	0.409921	12	Very High	BCmicrO|BiTargeting
ADGRG2	Q8IZP9	hsa-miR-3	0.409795	12	Very High	BCmicrO|CoMeTa|DI
ASB6	Q9NWX5	hsa-miR-3	0.408874	10	Very High	BCmicrO|CoMeTa|El
TMEM41A	Q96HV5	hsa-miR-3	0.408736	11	Very High	BCmicrO|BiTargeting
HECW1	Q76N89	hsa-miR-3	0.408393	12	Very High	BCmicrO|DIANA|ElMI
ACVR1	Q04771	hsa-miR-3	0.408345	12	Very High	BCmicrO|CoMeTa|Cu
NFIC	P08651	hsa-miR-3	0.408146	12	Very High	BCmicrO|BiTargeting
MKNK1	Q9BUB5	hsa-miR-3	0.407893	14	Very High	BCmicrO|BiTargeting
PIEZO2	Q9H5I5	hsa-miR-3	0.407884	14	Very High	BCmicrO|BiTargeting
PDGFD	Q9GZP0	hsa-miR-3	0.4077	12	Very High	BCmicrO|DIANA|ElMI
C8orf86	Q6ZUL3	hsa-miR-3	0.407437	12	Very High	BCmicrO|DIANA|ElMI
USP24	Q9UPU5	hsa-miR-3	0.407146	13	Very High	BCmicrO|DIANA|ElMI
ADAM10	O14672	hsa-miR-3	0.406731	12	Very High	BCmicrO|BiTargeting
FAM204A	Q9H8W3	hsa-miR-3	0.406477	12	Very High	BCmicrO|CoMeTa|DI
SELENON	Q9NZV5	hsa-miR-3	0.406268	13	Very High	BCmicrO|BiTargeting
RBM33	Q96EV2	hsa-miR-3	0.406259	13	Very High	BCmicrO|DIANA|ElMI
SLC39A1	Q9NY26	hsa-miR-3	0.405982	14	Very High	BCmicrO|BiTargeting
KCMF1	Q9P0J7	hsa-miR-3	0.405837	9	Very High	BCmicrO|BiTargeting
SEMA6A	Q9H2E6	hsa-miR-3	0.405765	12	Very High	BCmicrO|BiTargeting
RHD	Q02161	hsa-miR-3	0.404701	13	Very High	BCmicrO|CoMeTa|DI
GRIN2A	Q12879	hsa-miR-3	0.404654	11	Very High	BCmicrO|CoMeTa|DI
HOXB9	P17482	hsa-miR-3	0.404602	12	Very High	BCmicrO|DIANA|ElMI
ZNF592	Q92610	hsa-miR-3	0.404121	13	Very High	BCmicrO|BiTargeting
FAM196A	Q6ZSG2	hsa-miR-3	0.403938	12	Very High	BCmicrO|BiTargeting
IGF2BP1	Q9NZI8	hsa-miR-3	0.403873	9	Very High	BCmicrO|ElMMo3|ME
CAV2	P51636	hsa-miR-3	0.403485	11	Very High	BCmicrO|DIANA|ElMI
CRELD1	Q96HD1	hsa-miR-3	0.403384	12	Very High	BCmicrO|BiTargeting
NCOR2	Q9Y618	hsa-miR-3	0.40338	11	Very High	BCmicrO|BiTargeting
ZNF337	Q9Y3M9	hsa-miR-3	0.402807	13	Very High	BCmicrO|BiTargeting
ZNF362	Q5T0B9	hsa-miR-3	0.401961	11	Very High	BCmicrO|CoMeTa|DI
CRKL	P46109	hsa-miR-3	0.401923	12	Very High	BCmicrO|BiTargeting
KLK2	P20151	hsa-miR-3	0.401877	13	Very High	BCmicrO|BiTargeting
SLF2	Q8IX21	hsa-miR-3	0.401624	12	Very High	BCmicrO|DIANA|ElMI
BET1	O15155	hsa-miR-3	0.401222	11	Very High	BCmicrO|CoMeTa|El
ATG2B	Q96BY7	hsa-miR-3	0.401151	12	Very High	BCmicrO|CoMeTa|DI
AFF2	P51816	hsa-miR-3	0.400404	13	Very High	BCmicrO|BiTargeting
SPDYE1	Q8NFV5	hsa-miR-3	0.400279	11	Very High	BCmicrO|DIANA|ElMI
ADARB1	P78563	hsa-miR-3	0.400275	11	Very High	BCmicrO|CoMeTa|DI
KLF14	Q8TD94	hsa-miR-3	0.39931	13	Very High	BCmicrO|BiTargeting
TENM1	Q9UKZ4	hsa-miR-3	0.39927	13	Very High	BCmicrO|CoMeTa|DI
SELENOK	Q9Y6D0	hsa-miR-3	0.398756	8	Very High	BCmicrO|BiTargeting
WHRN	Q9P202	hsa-miR-3	0.398651	13	Very High	BCmicrO|BiTargeting
LIN28B	Q6ZN17	hsa-miR-3	0.397668	11	Very High	BCmicrO|DIANA|ElMI
CYTH3	O43739	hsa-miR-3	0.397568	12	Very High	BCmicrO|CoMeTa|DI
TIMM13	Q9Y5L4	hsa-miR-3	0.397447	13	Very High	BCmicrO|BiTargeting
TSC22D4	Q9Y3Q8	hsa-miR-3	0.397404	13	Very High	BCmicrO|BiTargeting
ANTXR1	Q9H6X2	hsa-miR-3	0.396581	14	Very High	BCmicrO|DIANA|ElMI
ZIK1	Q3SY52	hsa-miR-3	0.396296	10	Very High	BCmicrO|DIANA|ElMI
POU4F2	Q12837	hsa-miR-3	0.396295	13	Very High	BCmicrO|DIANA|ElMI
ALDH1A2	O94788	hsa-miR-3	0.395575	12	Very High	BCmicrO|DIANA|ElMI
KSR2	Q6VAB6	hsa-miR-3	0.395552	10	Very High	BCmicrO|DIANA|ElMI
RAB36	O95755	hsa-miR-3	0.395406	12	Very High	BCmicrO|DIANA|ElMI

TRIM14	Q14142	hsa-miR-3	0.395274	12	Very High	BCmicrO|DIANA|EIMI
FAM220A	Q7Z4H9	hsa-miR-3	0.395175	13	Very High	BCmicrO|DIANA|EIMI
GLIPR2	Q9H4G4	hsa-miR-3	0.394896	12	Very High	BCmicrO|BiTargeting
TUSC2	O75896	hsa-miR-3	0.394812	10	Very High	BCmicrO|CoMeTa|EIM
SLC46A2	Q9BY10	hsa-miR-3	0.393952	12	Very High	BCmicrO|DIANA|EIMI
NT5DC3	Q86UY8	hsa-miR-3	0.393644	13	Very High	BCmicrO|CoMeTa|DIA
CNTLN	Q9NXG0	hsa-miR-3	0.393334	14	Very High	BCmicrO|BiTargeting
ADCY1	Q08828	hsa-miR-3	0.3927	11	Very High	BCmicrO|BiTargeting
ZC3HAV1	Q7Z2W4	hsa-miR-3	0.39125	12	Very High	BCmicrO|DIANA|EIMI
CNNM1	Q9NRU3	hsa-miR-3	0.39119	12	Very High	BCmicrO|BiTargeting
GINS3	Q9BRX5	hsa-miR-3	0.390756	13	Very High	BCmicrO|BiTargeting
SUV39H1	O43463	hsa-miR-3	0.390466	11	Very High	BCmicrO|DIANA|EIMI
CREB1	P16220	hsa-miR-3	0.390396	10	Very High	BCmicrO|DIANA|EIMI
PCP4L1	A6NKN8	hsa-miR-3	0.390247	10	Very High	BCmicrO|DIANA|EIMI
SLC7A5	Q01650	hsa-miR-3	0.390128	11	Very High	BCmicrO|DIANA|EIMI
CDC14B	O60729	hsa-miR-3	0.389493	12	Very High	BCmicrO|BiTargeting
MTMR3	Q13615	hsa-miR-3	0.389136	11	Very High	BCmicrO|BiTargeting
FAM110A	Q9BQ89	hsa-miR-3	0.389114	14	Very High	BCmicrO|BiTargeting
ZER1	Q7Z7L7	hsa-miR-3	0.389081	12	Very High	BCmicrO|BiTargeting
PSAP	P07602	hsa-miR-3	0.389049	12	Very High	BCmicrO|DIANA|EIMI
ATP11A	P98196	hsa-miR-3	0.388934	13	Very High	BCmicrO|BiTargeting
GPR4	P46093	hsa-miR-3	0.388633	11	Very High	BCmicrO|BiTargeting
MAPK1	P28482	hsa-miR-3	0.388502	12	Very High	BCmicrO|BiTargeting
PEA15	Q15121	hsa-miR-3	0.388342	11	Very High	BCmicrO|CoMeTa|DIA
ZNF423	Q2M1K9	hsa-miR-3	0.388226	10	Very High	BCmicrO|BiTargeting
CTNNA1	P35221	hsa-miR-3	0.388205	13	Very High	BCmicrO|BiTargeting
MAP6	Q96JE9	hsa-miR-3	0.387851	12	Very High	BCmicrO|DIANA|EIMI
SLC24A4	Q8NFF2	hsa-miR-3	0.387758	13	Very High	BCmicrO|BiTargeting
CEP350	Q5VT06	hsa-miR-3	0.387743	12	Very High	BCmicrO|Cupid|DIAN
PARPBP	Q9NWS1	hsa-miR-3	0.386796	12	Very High	BCmicrO|CoMeTa|DIA
TMEM30A	Q9NV96	hsa-miR-3	0.386637	12	Very High	BCmicrO|CoMeTa|DIA
IGSF3	O75054	hsa-miR-3	0.386604	13	Very High	BCmicrO|BiTargeting
GABRG1	Q8N1C3	hsa-miR-3	0.386116	12	Very High	BCmicrO|DIANA|EIMI
RALB	P11234	hsa-miR-3	0.386051	11	Very High	BCmicrO|DIANA|EIMI
PPIL6	Q8IXY8	hsa-miR-3	0.386046	11	Very High	BCmicrO|DIANA|EIMI
ABLIM2	Q6H8Q1	hsa-miR-3	0.385886	11	Very High	BCmicrO|BiTargeting
MYOM2	P54296	hsa-miR-3	0.385589	13	Very High	BCmicrO|BiTargeting
CERK	Q8TCT0	hsa-miR-3	0.38528	12	Very High	BCmicrO|DIANA|EIMI
ZNF526	Q8TF50	hsa-miR-3	0.385103	12	Very High	BCmicrO|BiTargeting
FSTL4	Q6MZW2	hsa-miR-3	0.384964	11	Very High	BCmicrO|CoMeTa|DIA
CACNA1I	Q9P0X4	hsa-miR-3	0.384956	12	Very High	BCmicrO|BiTargeting
ZNF286A	Q9HBT8	hsa-miR-3	0.38489	12	Very High	BCmicrO|DIANA|EIMI
ERICH3	Q5RHP9	hsa-miR-3	0.384017	10	Very High	BCmicrO|EIMMo3|MA
HSPG2	P98160	hsa-miR-3	0.383285	12	Very High	BCmicrO|BiTargeting
STX4	Q12846	hsa-miR-3	0.382768	11	Very High	BCmicrO|BiTargeting
DGKB	Q9Y6T7	hsa-miR-3	0.382747	12	Very High	BCmicrO|BiTargeting
NFE2L1	Q14494	hsa-miR-3	0.38259	11	Very High	BCmicrO|DIANA|EIMI

|CoMeTa|Cupid|DIANA|ElMMo3|MBStar|MAMI|microrna.org|MirAncesTar|miRDB|MirMAP|MirTa
|CoMeTa|DIANA|ElMMo3|MAMI|microrna.org|MirAncesTar|MirMAP|MirTar|miRTar2GO|Mirza-G
ANA|ElMMo3|MAMI|microrna.org|MirAncesTar|mirbase|miRDB|MirMAP|MirTar|Mirza-G|PACCM
|CoMeTa|DIANA|ElMMo3|MBStar|microrna.org|MirAncesTar|miRDB|MirMAP|MirTar|miRTar2GO|
|CoMeTa|DIANA|ElMMo3|MBStar|MAMI|microrna.org|MirAncesTar|mirbase|miRDB|MirMAP|Mir
ANA|ElMMo3|MBStar|MAMI|microrna.org|MirAncesTar|miRDB|MirMAP|Mirza-G|MultiMiTar|PAC
|CoMeTa|DIANA|ElMMo3|microrna.org|MirAncesTar|miRDB|MirMAP|MirTar|Mirza-G|MultiMiTar
|CoMeTa|DIANA|ElMMo3|MBStar|MAMI|microrna.org|MirAncesTar|miRDB|MirMAP|miRTar2GO|
|CoMeTa|DIANA|ElMMo3|MBStar|microrna.org|MirAncesTar|miRDB|MirMAP|MirTar|miRTar2GO|
ANA|ElMMo3|MBStar|microrna.org|MirAncesTar|miRDB|MirMAP|MirTar|miRTar2GO|Mirza-G|PA
|CoMeTa|DIANA|ElMMo3|MBStar|microrna.org|MirAncesTar|mirbase|miRDB|MirMAP|MirTar|miR
|CoMeTa|DIANA|ElMMo3|MAMI|microrna.org|MirAncesTar|miRDB|MirMAP|Mirza-G|PACCMIT|R
|CoMeTa|Cupid|DIANA|ElMMo3|MBStar|microrna.org|MirAncesTar|miRDB|MirMAP|MirTar|Mirza
|CoMeTa|DIANA|ElMMo3|microrna.org|MirAncesTar|miRDB|MirMAP|MirTar|Mirza-G|PACCMIT|R
|DIANA|ElMMo3|MAMI|microrna.org|MirAncesTar|mirbase|miRDB|MirMAP|MirSNP|MirTar|Mirza
|CoMeTa|DIANA|ElMMo3|MAMI|microrna.org|MirAncesTar|miRDB|MirMAP|MirTar|RNAhybrid|Ta
pid|DIANA|ElMMo3|MAMI|microrna.org|MirAncesTar|miRDB|MirMAP|MirSNP|miRTar2GO|Mirza
|DIANA|ElMMo3|MBStar|microrna.org|MirAncesTar|miRDB|MirMAP|MirSNP|miRTar2GO|Mirza-G
ANA|ElMMo3|MBStar|microrna.org|MirAncesTar|miRDB|MirMAP|miRTar2GO|Mirza-G|PACCMIT|
|CoMeTa|DIANA|ElMMo3|MBStar|microrna.org|MirAncesTar|miRDB|MirMAP|Mirza-G|MultiMiTa
|CoMeTa|DIANA|ElMMo3|MBStar|microrna.org|MirAncesTar|miRDB|MirMAP|MirTar|Mirza-G|Rep
ANA|ElMMo3|microrna.org|MirAncesTar|miRDB|MirMAP|MirSNP|MirTar|miRTar2GO|Mirza-G|Re
|CoMeTa|DIANA|ElMMo3|microrna.org|MirAncesTar|miRDB|MirMAP|MirTar|Mirza-G|MultiMiTar
ANA|ElMMo3|microrna.org|MirAncesTar|miRDB|MirMAP|Mirza-G|MultiMiTar|RepTar|RNAhybrid
|CoMeTa|DIANA|ElMMo3|MBStar|microrna.org|MirAncesTar|miRDB|MirMAP|MultiMiTar|PACCM
|CoMeTa|DIANA|ElMMo3|MAMI|microrna.org|MirAncesTar|mirbase|miRDB|MirMAP|Mirza-G|PA
|DIANA|ElMMo3|MBStar|microrna.org|MirAncesTar|miRDB|MirMAP|Mirza-G|MultiMiTar|RepTar|
IA|ElMMo3|MBStar|microrna.org|MirAncesTar|miRDB|MirMAP|miRTar2GO|MultiMiTar|TargetRan
pid|DIANA|ElMMo3|MBStar|microrna.org|MirAncesTar|MirMAP|Mirza-G|PACCMIT|RepTar|RNA2
ANA|ElMMo3|microrna.org|MirAncesTar|miRDB|MirMAP|Mirza-G|PACCMIT|RNAhybrid|TargetRa
Mo3|microrna.org|MirAncesTar|miRDB|MirMAP|MirTar|Mirza-G|RNAhybrid|TargetRank|TargetSc
Mo3|microrna.org|MirAncesTar|mirbase|miRDB|MirMAP|miRTar2GO|Mirza-G|RepTar|RNAhybrid
Mo3|MBStar|microrna.org|MirAncesTar|miRDB|MirMAP|MirTar|Mirza-G|TargetRank|TargetScan|
pid|DIANA|ElMMo3|MBStar|microrna.org|MirAncesTar|miRDB|MirMAP|MirTar|miRTar2GO|Mirza
ANA|ElMMo3|microrna.org|MirAncesTar|miRDB|MirMAP|miRTar2GO|PACCMIT|RNAhybrid|Targe
|CoMeTa|DIANA|ElMMo3|MAMI|MirAncesTar|miRDB|MirMAP|RepTar|RNAhybrid|TargetScan
|CoMeTa|DIANA|ElMMo3|MBStar|MirAncesTar|miRDB|MirMAP|MirSNP|miRTar2GO|Mirza-G|Mul
pid|DIANA|ElMMo3|MBStar|microrna.org|MirAncesTar|MirMAP|miRTar2GO|Mirza-G|MultiMiTar
|DIANA|ElMMo3|MAMI|MirAncesTar|miRDB|MirMAP|MirSNP|miRTar2GO|Mirza-G|MultiMiTar|R
IA|ElMMo3|MAMI|microrna.org|MirAncesTar|mirbase|MirMAP|MirTar|Mirza-G|RNAhybrid|Targe
ANA|ElMMo3|MBStar|MAMI|MirAncesTar|miRDB|MirMAP|MirTar|Mirza-G|RNAhybrid|TargetRan
ANA|ElMMo3|microrna.org|MirAncesTar|miRDB|MirMAP|Mirza-G|RepTar|RNA22|RNAhybrid|Tar
|CoMeTa|ElMMo3|MBStar|MirAncesTar|miRDB|miRTar2GO|Mirza-G|MultiMiTar|RepTar|TargetRa
|DIANA|ElMMo3|MBStar|MAMI|microrna.org|MirAncesTar|miRDB|MirMAP|MirTar|miRTar2GO|Mi
|CoMeTa|DIANA|ElMMo3|MBStar|microrna.org|MirAncesTar|MirMAP|miRTar2GO|Mirza-G|Multi

|CoMeTa|DIANA|ElMMo3|MirAncesTar|MirMAP|miRTar2GO|Mirza-G|MultiMiTar|RepTar|TargetR
|CoMeTa|DIANA|ElMMo3|microrna.org|MirAncesTar|miRDB|MirMAP|miRTar2GO|Mirza-G|RepTa
ANA|ElMMo3|microrna.org|MirAncesTar|miRDB|MirMAP|miRTar2GO|Mirza-G|MultiMiTar|RNAhy
|CoMeTa|DIANA|ElMMo3|MBStar|microrna.org|MirAncesTar|miRDB|MirMAP|Mirza-G|PACCMIT|
ANA|ElMMo3|microrna.org|MirAncesTar|MirMAP|Mirza-G|RNA22|RNAhybrid|TargetRank|Target
|CoMeTa|DIANA|ElMMo3|MBStar|MirAncesTar|miRDB|MirMAP|miRTar2GO|Mirza-G|MultiMiTar|
|DIANA|ElMMo3|MBStar|microrna.org|MirAncesTar|miRDB|MirMAP|Mirza-G|MultiMiTar|PACCM
ANA|ElMMo3|microrna.org|MirAncesTar|miRDB|MirMAP|MirTar|miRTar2GO|Mirza-G|RNAhybrid
ANA|ElMMo3|MBStar|microrna.org|MirAncesTar|miRDB|MirMAP|MirTar|Mirza-G|MultiMiTar|Tar
|DIANA|ElMMo3|microrna.org|MirAncesTar|miRDB|MirMAP|Mirza-G|MultiMiTar|RepTar|RNAhyb
|CoMeTa|ElMMo3|MBStar|microrna.org|MirAncesTar|mirbase|miRDB|MirMAP|MirTar|Mirza-G|Re
|DIANA|ElMMo3|microrna.org|MirAncesTar|miRDB|MirMAP|MirTar|Mirza-G|RepTar|RNA22|Targ
|DIANA|ElMMo3|MBStar|microrna.org|MirAncesTar|miRDB|MirMAP|MirSNP|MirTar|Mirza-G|Mul
|DIANA|ElMMo3|MBStar|MirAncesTar|miRDB|MirMAP|MirSNP|MirTar|miRTar2GO|Mirza-G|PACC
|DIANA|ElMMo3|microrna.org|MirAncesTar|miRDB|MirMAP|MirTar|Mirza-G|TargetRank|TargetS
Mo3|microrna.org|MirAncesTar|miRDB|MirMAP|MirTar|Mirza-G|RNAhybrid|TargetRank|TargetSc
Mo3|microrna.org|MirAncesTar|miRDB|MirMAP|Mirza-G|MultiMiTar|TargetRank|TargetScan|Targ
|CoMeTa|DIANA|ElMMo3|MirAncesTar|miRDB|MirMAP|Mirza-G|MultiMiTar|PACCMIT|RepTar|Ta
|DIANA|ElMMo3|MBStar|microrna.org|MirAncesTar|miRDB|MirMAP|MirTar|Mirza-G|RepTar|RNA
|CoMeTa|DIANA|ElMMo3|microrna.org|MirAncesTar|miRDB|MirMAP|miRTar2GO|Mirza-G|PACC
|CoMeTa|DIANA|ElMMo3|MAMI|MirAncesTar|mirbase|miRDB|MirMAP|Mirza-G|PACCMIT|RepTa
|DIANA|ElMMo3|MBStar|MAMI|MirAncesTar|mirbase|miRDB|MirMAP|MirSNP|Mirza-G|PACCMIT
|DIANA|ElMMo3|microrna.org|MirAncesTar|miRDB|MirMAP|MirTar|miRTar2GO|Mirza-G|RepTar|
ANA|ElMMo3|microrna.org|MirAncesTar|miRDB|MirMAP|MirTar|Mirza-G|PACCMIT|RNAhybrid|T
Mo3|microrna.org|MirAncesTar|miRDB|MirMAP|MirTar|miRTar2GO|RepTar|RNAhybrid|TargetRan
ANA|ElMMo3|microrna.org|MirAncesTar|miRDB|MirMAP|MirSNP|Mirza-G|RepTar|RNAhybrid|Tar
|DIANA|ElMMo3|MirAncesTar|miRDB|MirMAP|MirTar|Mirza-G|PACCMIT|RepTar|RNAhybrid|Targ
|DIANA|ElMMo3|MirAncesTar|miRDB|MirMAP|miRTar2GO|Mirza-G|PACCMIT|RepTar|RNAhybrid
|DIANA|ElMMo3|microrna.org|MirAncesTar|miRDB|MirMAP|MirTar|RepTar|RNAhybrid|TargetRan
ANA|ElMMo3|microrna.org|MirAncesTar|miRDB|MirMAP|miRTar2GO|Mirza-G|RepTar|RNAhybri
Mo3|MBStar|microrna.org|MirAncesTar|miRDB|MirMAP|MirTar|Mirza-G|RepTar|TargetRank|Targ
ANA|ElMMo3|MBStar|microrna.org|MirAncesTar|miRDB|MirMAP|MultiMiTar|RepTar|TargetRank|
Mo3|microrna.org|MirAncesTar|miRDB|MirMAP|MirTar|miRTar2GO|Mirza-G|MultiMiTar|PACCMI
ANA|ElMMo3|MBStar|microrna.org|MirAncesTar|miRDB|MirMAP|MirSNP|miRTar2GO|Mirza-G|M
ANA|ElMMo3|microrna.org|MirAncesTar|miRDB|MirMAP|MirTar|miRTar2GO|Mirza-G|RepTar|RN
|CoMeTa|DIANA|ElMMo3|MBStar|microrna.org|MirAncesTar|mirbase|miRDB|MirMAP|PACCMIT|
|CoMeTa|DIANA|ElMMo3|microrna.org|MirAncesTar|miRDB|MirMAP|MirSNP|MirTar|MultiMiTar|
|DIANA|ElMMo3|microrna.org|MirAncesTar|mirbase|miRDB|MirMAP|MirTar|RepTar|RNAhybrid|T
|CoMeTa|DIANA|ElMMo3|microrna.org|MirAncesTar|miRDB|MirMAP|miRTar2GO|Mirza-G|RepTa
|Cupid|DIANA|ElMMo3|MBStar|microrna.org|MirAncesTar|miRDB|MirMAP|miRTar2GO|Mirza-G|
|CoMeTa|Cupid|DIANA|ElMMo3|MBStar|microrna.org|MirAncesTar|mirbase|miRDB|MirMAP|Mirz
|CoMeTa|DIANA|ElMMo3|MBStar|microrna.org|MirAncesTar|miRDB|MirMAP|MirSNP|MirTar|Mirz
|DIANA|ElMMo3|microrna.org|MirAncesTar|miRDB|MirMAP|Mirza-G|RepTar|RNA22|TargetRank|
Mo3|microrna.org|MirAncesTar|miRDB|MirMAP|MirTar|miRTar2GO|Mirza-G|RNAhybrid|TargetRa
|DIANA|ElMMo3|MBStar|MirAncesTar|miRDB|MirMAP|miRTar2GO|Mirza-G|MultiMiTar|PACCMIT
ANA|ElMMo3|MBStar|microrna.org|MirAncesTar|miRDB|MirMAP|MirTar|Mirza-G|MultiMiTar|Rep
|CoMeTa|DIANA|ElMMo3|MBStar|microrna.org|MirAncesTar|miRDB|MirMAP|miRTar2GO|Mirza-
|DIANA|ElMMo3|MBStar|MirAncesTar|MirMAP|miRTar2GO|Mirza-G|PACCMIT|RNAhybrid|Target
ANA|ElMMo3|MBStar|microrna.org|MirAncesTar|MirMAP|MirTar|Mirza-G|RNAhybrid|TargetRank
|DIANA|ElMMo3|microrna.org|MirAncesTar|mirbase|miRDB|MirMAP|MirSNP|MirTar|Mirza-G|Rep
|CoMeTa|DIANA|ElMMo3|MBStar|microrna.org|MirAncesTar|miRDB|MirMAP|MirTar|Mirza-G|Mu
|CoMeTa|ElMMo3|MBStar|microrna.org|MirAncesTar|miRDB|MirMAP|MirTar|miRTar2GO|Mirza-
Mo3|microrna.org|MirAncesTar|miRDB|MirMAP|MirTar|Mirza-G|RepTar|RNAhybrid|TargetScan
|CoMeTa|DIANA|ElMMo3|microrna.org|MirAncesTar|miRDB|MirMAP|MirSNP|Mirza-G|MultiMiTa
|CoMeTa|DIANA|ElMMo3|microrna.org|MirAncesTar|miRDB|MirMAP|MirSNP|Mirza-G|MultiMiTa
|CoMeTa|DIANA|ElMMo3|MBStar|microrna.org|MirAncesTar|miRDB|MirMAP|MirSNP|MultiMiTar|
ANA|ElMMo3|microrna.org|MirAncesTar|miRDB|MirMAP|MirSNP|miRTar2GO|Mirza-G|RNAhybri
Mo3|MBStar|MirAncesTar|miRDB|MirMAP|miRTar2GO|Mirza-G|PACCMIT|RNAhybrid|TargetScan

|DIANA|EIMMo3|MBStar|microrna.org|MirAncesTar|miRDB|MirMAP|MirTar|Mirza-G|RepTar|RNA
ANA|EIMMo3|microrna.org|MirAncesTar|mirbase|MirMAP|miRTar2GO|Mirza-G|RepTar|RNAhybr
IA|EIMMo3|MBStar|microrna.org|MirAncesTar|MirMAP|miRTar2GO|Mirza-G|MultiMiTar|RNAhyb
|DIANA|EIMMo3|MAMI|MirAncesTar|mirbase|miRDB|MirMAP|miRTar2GO|MultiMiTar|RepTar|RN
|CoMeTa|DIANA|EIMMo3|microrna.org|MirAncesTar|miRDB|MirMAP|miRTar2GO|Mirza-G|RepTa
IA|EIMMo3|MBStar|MirAncesTar|miRDB|MirMAP|miRTar2GO|Mirza-G|MultiMiTar|RepTar|RNAhy
|DIANA|EIMMo3|MAMI|microrna.org|MirAncesTar|miRDB|MirMAP|MirSNP|MirTar|Mirza-G|RepT
ANA|EIMMo3|microrna.org|MirAncesTar|miRDB|MirMAP|RepTar|RNAhybrid|TargetRank|TargetS
|DIANA|EIMMo3|microrna.org|MirAncesTar|miRDB|MirMAP|MirTar|miRTar2GO|Mirza-G|RepTar|
Mo3|microrna.org|MirAncesTar|miRDB|MirMAP|MirTar|miRTar2GO|Mirza-G|RepTar|TargetRank|
|DIANA|EIMMo3|MBStar|microrna.org|MirAncesTar|miRDB|MirMAP|miRTar2GO|Mirza-G|PACCM
|DIANA|EIMMo3|MAMI|microrna.org|MirAncesTar|miRDB|MirMAP|MirTar|Mirza-G|RepTar|RNAh
|CoMeTa|DIANA|EIMMo3|MBStar|microrna.org|MirAncesTar|mirbase|miRDB|MirMAP|MirTar|Mir
ANA|EIMMo3|microrna.org|MirAncesTar|miRDB|MirMAP|MirSNP|miRTar2GO|Mirza-G|RepTar|Ta
|CoMeTa|DIANA|EIMMo3|microrna.org|MirAncesTar|MirMAP|miRTar2GO|Mirza-G|MultiMiTar|P
Mo3|microrna.org|MirAncesTar|miRDB|MirMAP|MirTar|miRTar2GO|Mirza-G|RepTar|RNAhybrid|
|CoMeTa|DIANA|EIMMo3|microrna.org|MirAncesTar|miRDB|MirMAP|Mirza-G|MultiMiTar|PACCM
|DIANA|EIMMo3|MirAncesTar|miRDB|MirMAP|MirTar|miRTar2GO|Mirza-G|RepTar|TargetRank|Ta
ANA|EIMMo3|microrna.org|MirAncesTar|miRDB|MirMAP|Mirza-G|RepTar|RNAhybrid|TargetScan
ANA|EIMMo3|MBStar|MirAncesTar|miRDB|MirMAP|MirTar|Mirza-G|RepTar|RNAhybrid|TargetRar
MMo3|MirAncesTar|miRDB|MirMAP|Mirza-G|MultiMiTar|RepTar|RNAhybrid|TargetRank|TargetSc
IA|EIMMo3|MBStar|microrna.org|MirMAP|Mirza-G|RepTar|RNAhybrid|TargetScan|TargetSpy
|CoMeTa|DIANA|EIMMo3|MBStar|microrna.org|MirAncesTar|miRDB|MirMAP|miRTar2GO|Mirza-
ANA|EIMMo3|microrna.org|MirAncesTar|miRDB|MirMAP|miRTar2GO|Mirza-G|MultiMiTar|RepTar
Mo3|MAMI|microrna.org|MirAncesTar|MirMAP|MirTar|Mirza-G|RNAhybrid|TargetRank
|DIANA|EIMMo3|microrna.org|MirAncesTar|miRDB|MirMAP|MirTar|miRTar2GO|Mirza-G|RNAhyb
|DIANA|EIMMo3|MBStar|MAMI|MirAncesTar|mirbase|miRDB|MirMAP|Mirza-G|RepTar|RNAhybri

ANA|EIMMo3|microrna.org|MirAncesTar|miRDB|MirMAP|Mirza-G|MultiMiTar|RNAhybrid|TargetR
|CoMeTa|DIANA|EIMMo3|microrna.org|MirAncesTar|miRDB|MirMAP|miRTar2GO|Mirza-G|RepTa
Mo3|MBStar|MAMI|microrna.org|MirAncesTar|miRDB|MirMAP|MirTar|Mirza-G|RepTar|RNA22|RN
ANA|EIMMo3|microrna.org|MirAncesTar|miRDB|MirMAP|miRTar2GO|Mirza-G|TargetRank|Target
|CoMeTa|DIANA|EIMMo3|microrna.org|MirAncesTar|mirbase|miRDB|MirMAP|miRTar2GO|Mirza-
|DIANA|EIMMo3|MBStar|microrna.org|MirAncesTar|miRDB|MirMAP|MirTar|miRTar2GO|Mirza-G|
ANA|EIMMo3|MBStar|MirAncesTar|miRDB|MirMAP|MirSNP|miRTar2GO|PACCMIT|RNAhybrid|Tar
ANA|EIMMo3|MBStar|MirAncesTar|miRDB|MirMAP|Mirza-G|RNAhybrid|TargetScan|TargetSpy
|DIANA|EIMMo3|MBStar|microrna.org|MirAncesTar|mirbase|miRDB|MirMAP|Mirza-G|RNAhybrid
ANA|EIMMo3|MBStar|microrna.org|MirAncesTar|miRDB|MirMAP|MirTar|RepTar|RNAhybrid|Targe
|CoMeTa|DIANA|EIMMo3|microrna.org|MirAncesTar|miRDB|MirMAP|Mirza-G|RepTar|RNAhybric
AMI|microrna.org|MirAncesTar|mirbase|miRDB|MirMAP|MirSNP|Mirza-G|RepTar|RNAhybrid|Targ
Mo3|MBStar|microrna.org|MirAncesTar|miRDB|MirMAP|Mirza-G|RNAhybrid|TargetRank|TargetS
ANA|EIMMo3|microrna.org|MirAncesTar|miRDB|MirMAP|MirTar|Mirza-G|RNAhybrid|TargetRank
Mo3|microrna.org|MirAncesTar|miRDB|MirMAP|MirTar|miRTar2GO|Mirza-G|RNAhybrid|TargetRa
ANA|EIMMo3|microrna.org|MirAncesTar|miRDB|MirMAP|RNAhybrid|TargetRank|TargetSpy
ANA|EIMMo3|MBStar|microrna.org|MirAncesTar|miRDB|MirMAP|Mirza-G|MultiMiTar|PACCMIT|R
ANA|EIMMo3|microrna.org|MirAncesTar|miRDB|MirMAP|MirTar|RNAhybrid|TargetRank|TargetSp
Mo3|MAMI|microrna.org|MirAncesTar|mirbase|miRDB|MirMAP|Mirza-G|PACCMIT|RepTar|RNAhy
|CoMeTa|DIANA|EIMMo3|microrna.org|MirAncesTar|miRTar2GO|PACCMIT|RNAhybrid|TargetRa
|DIANA|EIMMo3|MBStar|microrna.org|MirAncesTar|miRDB|miRTar2GO|Mirza-G|RepTar|TargetRa
ANA|EIMMo3|MBStar|microrna.org|MirAncesTar|miRDB|MirMAP|MirSNP|miRTar2GO|Mirza-G|Ta
IA|EIMMo3|MirAncesTar|MirMAP|miRTar2GO|Mirza-G|RNAhybrid|TargetScan|TargetSpy
|CoMeTa|DIANA|EIMMo3|MirAncesTar|miRDB|MirMAP|MultiMiTar|PACCMIT|RNA22|RNAhybrid|
|CoMeTa|DIANA|EIMMo3|microrna.org|MirAncesTar|miRDB|MirMAP|miRTar2GO|Mirza-G|RepTa
|CoMeTa|DIANA|EIMMo3|microrna.org|MirAncesTar|miRDB|MirMAP|Mirza-G|RepTar|RNAhybric
|CoMeTa|DIANA|EIMMo3|MBStar|MirAncesTar|MirMAP|miRTar2GO|MultiMiTar|RNAhybrid|Targe
Mo3|MBStar|microrna.org|MirAncesTar|miRDB|MirMAP|Mirza-G|RNAhybrid|TargetRank|TargetS

|DIANA|EIMMo3|microrna.org|MirAncesTar|miRDB|MirMAP|miRTar2GO|Mirza-G|RepTar|RNA22|

Mo3|MBStar|microrna.org|MirAncesTar|miRDB|MirMAP|MirTar|Mirza-G|RNAhybrid|TargetRank|T
ANA|ElMMo3|microrna.org|MirAncesTar|miRDB|MirMAP|MirSNP|Mirza-G|RNA22|TargetRank
|DIANA|ElMMo3|MirAncesTar|miRDB|MirMAP|MirTar|miRTar2GO|Mirza-G|RepTar|RNAhybrid|Ta
ANA|ElMMo3|microrna.org|MirAncesTar|miRDB|MirMAP|miRTar2GO|Mirza-G|RepTar|RNAhybri
ANA|ElMMo3|MBStar|microrna.org|MirAncesTar|miRDB|MirMAP|Mirza-G|MultiMiTar|RNAhybrid
ANA|ElMMo3|MBStar|microrna.org|MirAncesTar|miRDB|MirMAP|miRTar2GO|Mirza-G|RepTar|RN
pid|ElMMo3|MBStar|MirAncesTar|MirMAP|Mirza-G|MultiMiTar|RNAhybrid|TargetScan|TargetSp
Mo3|microrna.org|MirAncesTar|mirbase|miRDB|MirMAP|Mirza-G|TargetScan|TargetSpy
ANA|ElMMo3|MBStar|microrna.org|MirAncesTar|mirbase|miRDB|MirMAP|MirSNP|RNAhybrid|Tar
|CoMeTa|DIANA|ElMMo3|microrna.org|MirAncesTar|miRDB|MirMAP|miRTar2GO|Mirza-G|RNAh
|CoMeTa|DIANA|ElMMo3|MirAncesTar|mirbase|miRDB|MirMAP|miRTar2GO|Mirza-G|RepTar|RN
|CoMeTa|DIANA|ElMMo3|MirAncesTar|miRDB|MirMAP|MirSNP|miRTar2GO|RepTar|RNAhybrid|T

A|ElMMo3|MBStar|microrna.org|MirAncesTar|MirMAP|miRTar2GO|Mirza-G|MultiMiTar|RNAhyb
Mo3|MBStar|microrna.org|MirAncesTar|miRDB|MirMAP|miRTar2GO|Mirza-G|RNAhybrid|TargetR
Mo3|microrna.org|MirAncesTar|miRDB|MirMAP|MirTar|Mirza-G|RNA22|RNAhybrid|TargetRank|T

|DIANA|ElMMo3|MBStar|microrna.org|MirAncesTar|miRDB|MirMAP|MirTar|Mirza-G|RepTar|RNA
|DIANA|ElMMo3|MBStar|microrna.org|MirAncesTar|miRDB|MirMAP|miRTar2GO|Mirza-G|RepTar
|DIANA|ElMMo3|microrna.org|MirAncesTar|miRDB|MirMAP|miRTar2GO|Mirza-G|RepTar|RNAhy
|CoMeTa|DIANA|ElMMo3|microrna.org|MirAncesTar|miRDB|MirMAP|Mirza-G|PACCMIT|RNAhyb
Mo3|microrna.org|MirAncesTar|miRDB|MirMAP|miRTar2GO|Mirza-G|RNAhybrid|TargetRank|Tar
Mo3|MBStar|microrna.org|MirAncesTar|miRDB|MirMAP|miRTar2GO|Mirza-G|MultiMiTar|TargetR
Mo3|MBStar|microrna.org|MirAncesTar|miRDB|MirMAP|MirTar|Mirza-G|RepTar|RNA22|RNAhyb
lMMo3|MBStar|microrna.org|MirAncesTar|miRDB|MirMAP|MirTar|miRTar2GO|Mirza-G|RNA22|Ta
|DIANA|ElMMo3|MBStar|microrna.org|miRDB|MirMAP|Mirza-G|PACCMIT|RepTar|TargetScan|Tar
|DIANA|ElMMo3|microrna.org|MirAncesTar|mirbase|miRDB|MirMAP|MirSNP|MirTar|Mirza-G|Tar
ANA|ElMMo3|microrna.org|MirAncesTar|miRDB|MirMAP|miRTar2GO|MultiMiTar|PACCMIT|RNAh
Mo3|microrna.org|MirAncesTar|mirbase|miRDB|MirMAP|MirSNP|miRTar2GO|Mirza-G|RNAhybri
|DIANA|ElMMo3|MBStar|microrna.org|MirAncesTar|miRDB|MirMAP|miRTar2GO|Mirza-G|PACCM
|CoMeTa|DIANA|ElMMo3|MBStar|microrna.org|MirAncesTar|miRDB|MirMAP|MirSNP|MirTar|Mirz
|DIANA|ElMMo3|MBStar|microrna.org|MirAncesTar|miRDB|MirMAP|MirSNP|miRTar2GO|Mirza-G
ANA|ElMMo3|MBStar|microrna.org|MirAncesTar|miRDB|MirMAP|MirTar|Mirza-G|RNAhybrid|Tar
|DIANA|ElMMo3|microrna.org|MirAncesTar|miRDB|MirMAP|miRTar2GO|Mirza-G|RNAhybrid|Tar
Mo3|MBStar|microrna.org|MirAncesTar|miRDB|MirMAP|MirTar|RNAhybrid|TargetRank|TargetSpy
|CoMeTa|DIANA|ElMMo3|microrna.org|MirAncesTar|miRDB|MirMAP|Mirza-G|RepTar|TargetRan
Mo3|MBStar|MirAncesTar|miRDB|MirMAP|miRTar2GO|RepTar|RNAhybrid|TargetRank|TargetScan
|DIANA|ElMMo3|MBStar|microrna.org|MirAncesTar|miRDB|MirMAP|miRTar2GO|Mirza-G|Target
|DIANA|ElMMo3|MBStar|microrna.org|MirAncesTar|MirMAP|miRTar2GO|MultiMiTar|RNAhybrid|
Mo3|MAMI|microrna.org|MirAncesTar|mirbase|miRDB|MirMAP|MirSNP|miRTar2GO|Mirza-G|Rep
|CoMeTa|DIANA|ElMMo3|microrna.org|MirAncesTar|miRDB|MirMAP|MirSNP|MirTar|Mirza-G|RN
|DIANA|ElMMo3|MBStar|microrna.org|MirAncesTar|miRDB|MirMAP|Mirza-G|PACCMIT|RepTar|R
|CoMeTa|DIANA|ElMMo3|MBStar|MirAncesTar|MirMAP|Mirza-G|MultiMiTar|RNAhybrid|TargetS
Mo3|MBStar|microrna.org|MirAncesTar|miRDB|MirMAP|Mirza-G|PACCMIT|RepTar|RNAhybrid|Ta

|DIANA|ElMMo3|MAMI|microrna.org|MirAncesTar|mirbase|miRDB|MirMAP|Mirza-G|RepTar|RNA
|DIANA|ElMMo3|MBStar|microrna.org|MirAncesTar|mirbase|miRDB|MirMAP|MirTar|Mirza-G|RN
|DIANA|ElMMo3|MirAncesTar|mirbase|miRDB|MirMAP|miRTar2GO|Mirza-G|RepTar|RNAhybrid|T
Mo3|MBStar|microrna.org|MirAncesTar|miRDB|MirMAP|MirSNP|MirTar|Mirza-G|RepTar|RNAhyb
Mo3|MBStar|microrna.org|MirAncesTar|miRDB|MirMAP|MirTar|Mirza-G|RepTar|RNAhybrid|Targe
|CoMeTa|DIANA|ElMMo3|microrna.org|MirAncesTar|mirbase|miRDB|MirMAP|RNAhybrid|Target
|CoMeTa|DIANA|ElMMo3|MBStar|microrna.org|MirAncesTar|miRDB|MirMAP|Mirza-G|RepTar|RN

|CoMeTa|DIANA|ElMMo3|MBStar|MirAncesTar|miRDB|MirMAP|MirSNP|MirTar|miRTar2GO|Mirza
Mo3|MBStar|MirAncesTar|MirMAP|miRTar2GO|Mirza-G|MultiMiTar|TargetRank|TargetSpy
|DIANA|ElMMo3|MirAncesTar|MirMAP|miRTar2GO|Mirza-G|MultiMiTar|RepTar|TargetScan
MMo3|microrna.org|MirAncesTar|miRDB|MirMAP|MirSNP|MirTar|RepTar|RNAhybrid|TargetRank|

|ElMMo3|MBStar|MirAncesTar|MirMAP|miRTar2GO|MultiMiTar|RepTar|RNAhybrid|TargetRank|Ta
|DIANA|ElMMo3|microrna.org|MirAncesTar|mirbase|miRDB|MirMAP|MirTar|miRTar2GO|Mirza-G
|DIANA|ElMMo3|MBStar|MirAncesTar|miRDB|MirMAP|miRTar2GO|Mirza-G|MultiMiTar|TargetSca
|DIANA|ElMMo3|MBStar|microrna.org|MirAncesTar|mirbase|miRDB|MirMAP|MirSNP|Mirza-G|Re
Mo3|MBStar|microrna.org|MirAncesTar|miRDB|MirMAP|miRTar2GO|Mirza-G|RepTar|RNAhybrid|
Mo3|MBStar|microrna.org|MirAncesTar|miRDB|MirMAP|miRTar2GO|Mirza-G|RepTar|RNAhybrid|

Mo3|MAMI|microrna.org|MirAncesTar|mirbase|miRDB|MirMAP|miRTar2GO|Mirza-G|RepTar|RNA
ANA|ElMMo3|MirAncesTar|miRDB|MirMAP|MirSNP|Mirza-G|MultiMiTar|RepTar|TargetRank
|DIANA|ElMMo3|MBStar|MirAncesTar|mirbase|miRDB|MirMAP|miRTar2GO|Mirza-G|RepTar|RNA
|DIANA|ElMMo3|MBStar|microrna.org|MirAncesTar|miRDB|MirMAP|Mirza-G|MultiMiTar|RepTar|
|DIANA|MBStar|microrna.org|MirAncesTar|MirMAP|miRTar2GO|RepTar|RNAhybrid|TargetScan
ANA|ElMMo3|microrna.org|MirMAP|miRTar2GO|Mirza-G|MultiMiTar|RNAhybrid|TargetSpy
MMo3|microrna.org|MirAncesTar|miRDB|MirMAP|MirTar|MultiMiTar|RNAhybrid|TargetSpy
Mo3|microrna.org|MirAncesTar|MirMAP|MirTar|Mirza-G|MultiMiTar|RNAhybrid|TargetRank
|DIANA|ElMMo3|MBStar|MAMI|microrna.org|MirAncesTar|mirbase|miRDB|MirMAP|miRTar2GO|
|CoMeTa|DIANA|ElMMo3|MBStar|microrna.org|MirAncesTar|MirMAP|Mirza-G|RNAhybrid|Target
|DIANA|ElMMo3|microrna.org|MirAncesTar|mirbase|miRDB|MirMAP|Mirza-G|RepTar|RNAhybrid
ANA|ElMMo3|MBStar|microrna.org|MirAncesTar|miRDB|MirMAP|MirSNP|Mirza-G|RNAhybrid|Ta
ANA|ElMMo3|microrna.org|MirAncesTar|mirbase|miRDB|MirMAP|RepTar|RNAhybrid|TargetRank
|CoMeTa|DIANA|ElMMo3|microrna.org|MirAncesTar|MirMAP|MirSNP|miRTar2GO|RNAhybrid|Ta
Mo3|MBStar|microrna.org|MirAncesTar|mirbase|miRDB|MirMAP|miRTar2GO|MultiMiTar|RNA22|
|DIANA|ElMMo3|MBStar|microrna.org|MirAncesTar|miRDB|MirMAP|MirSNP|miRTar2GO|Mirza-G
ANA|ElMMo3|MBStar|microrna.org|MirAncesTar|miRDB|MirMAP|MirSNP|Mirza-G|RNAhybrid|Ta
ANA|ElMMo3|MBStar|MirAncesTar|miRDB|MirMAP|miRTar2GO|Mirza-G|MultiMiTar|PACCMIT|RN
|CoMeTa|DIANA|ElMMo3|MBStar|MirAncesTar|miRDB|MirMAP|Mirza-G|PACCMIT|RepTar|RNAh
|CoMeTa|DIANA|ElMMo3|microrna.org|MirAncesTar|mirbase|miRDB|MirMAP|MirSNP|miRTar2G
Mo3|microrna.org|MirAncesTar|miRDB|MirMAP|MirTar|Mirza-G|RepTar|RNAhybrid|TargetRank|T
Mo3|microrna.org|MirAncesTar|miRDB|MirMAP|MirTar|Mirza-G|RNA22|RNAhybrid|TargetRank|T
|DIANA|ElMMo3|MAMI|microrna.org|MirAncesTar|mirbase|miRDB|MirMAP|MirSNP|Mirza-G|Rep
Mo3|MBStar|microrna.org|MirAncesTar|MirMAP|miRTar2GO|Mirza-G|MultiMiTar|RNAhybrid|Tar
ANA|ElMMo3|microrna.org|MirAncesTar|miRDB|MirMAP|Mirza-G|RepTar|RNAhybrid|TargetRank
ANA|ElMMo3|microrna.org|MirAncesTar|mirbase|miRDB|MirMAP|Mirza-G|PACCMIT|RepTar
Mo3|microrna.org|MirAncesTar|miRDB|MirMAP|MirSNP|Mirza-G|TargetRank|TargetSpy
|CoMeTa|DIANA|ElMMo3|MirAncesTar|MirMAP|miRTar2GO|Mirza-G|RepTar|RNAhybrid|TargetS
ANA|ElMMo3|microrna.org|MirAncesTar|miRDB|MirMAP|miRTar2GO|Mirza-G|TargetRank
Mo3|microrna.org|MirAncesTar|miRDB|MirMAP|MirTar|Mirza-G|RepTar|RNAhybrid|TargetSpy
|CoMeTa|ElMMo3|microrna.org|MirAncesTar|miRDB|MirMAP|MirSNP|miRTar2GO|RepTar|RNAhy
|DIANA|ElMMo3|microrna.org|MirAncesTar|miRDB|MirMAP|MirTar|Mirza-G|RepTar|RNAhybrid
Mo3|MBStar|MAMI|microrna.org|MirAncesTar|MirMAP|MultiMiTar|PACCMIT|RNAhybrid
ANA|ElMMo3|microrna.org|MirAncesTar|mirbase|miRDB|MirMAP|Mirza-G|RNAhybrid
|DIANA|ElMMo3|microrna.org|MirAncesTar|mirbase|miRDB|MirMAP|MirSNP|Mirza-G|RepTar|RN
|CoMeTa|DIANA|ElMMo3|MBStar|microrna.org|MirAncesTar|miRDB|MirMAP|miRTar2GO|Mirza-
|CoMeTa|DIANA|ElMMo3|MirAncesTar|miRDB|MirMAP|miRTar2GO|RepTar|RNAhybrid|TargetSca
|DIANA|ElMMo3|MBStar|microrna.org|MirAncesTar|miRDB|MirMAP|miRTar2GO|Mirza-G|RepTar
Mo3|microrna.org|MirAncesTar|miRDB|MirMAP|miRTar2GO|RepTar|RNAhybrid|TargetRank
|DIANA|ElMMo3|MirAncesTar|mirbase|miRDB|MirMAP|miRTar2GO|MultiMiTar|RepTar|TargetRar
|DIANA|MBStar|microrna.org|MirAncesTar|mirbase|miRDB|MirMAP|miRTar2GO|RNAhybrid|Targe
Mo3|MirAncesTar|MirMAP|miRTar2GO|Mirza-G|MultiMiTar|RepTar|RNAhybrid|TargetRank|Targe
|CoMeTa|DIANA|ElMMo3|microrna.org|MirAncesTar|mirbase|MirMAP|Mirza-G|MultiMiTar|RepTa
ANA|ElMMo3|microrna.org|MirAncesTar|miRDB|MirMAP|Mirza-G|RNAhybrid|TargetRank
ANA|ElMMo3|microrna.org|MirAncesTar|miRDB|MirMAP|miRTar2GO|Mirza-G|RNAhybrid
|DIANA|ElMMo3|microrna.org|MirAncesTar|mirbase|miRDB|MirMAP|miRTar2GO|Mirza-G|PACC
|DIANA|ElMMo3|MBStar|MirAncesTar|miRDB|MirMAP|miRTar2GO|MultiMiTar|RepTar|RNAhybric

|DIANA|ElMMo3|MAMI|microrna.org|MirAncesTar|MirMAP|miRTar2GO|Mirza-G|RepTar|RNAhyb
ANA|ElMMo3|MBStar|microrna.org|MirAncesTar|miRDB|MirMAP|miRTar2GO|Mirza-G|RepTar|RN

|CoMeTa|DIANA|ElMMo3|MBStar|MirAncesTar|miRDB|MirMAP|MultiMiTar|RepTar|TargetSpy
IA|ElMMo3|MBStar|microrna.org|MirAncesTar|MirMAP|Mirza-G|MultiMiTar|RNA22|TargetSpy
ANA|ElMMo3|microrna.org|MirAncesTar|miRDB|MirMAP|RepTar|RNAhybrid|TargetRank
Mo3|MBStar|microrna.org|MirAncesTar|miRDB|MirMAP|miRTar2GO|Mirza-G|TargetRank|TargetS
IA|ElMMo3|microrna.org|MirAncesTar|MirMAP|miRTar2GO|Mirza-G|RepTar|RNAhybrid
Mo3|microrna.org|MirAncesTar|mirbase|miRDB|MirMAP|RepTar|RNAhybrid|TargetRank|TargetSp
|DIANA|ElMMo3|MBStar|MirAncesTar|MirMAP|miRTar2GO|Mirza-G|MultiMiTar|RNAhybrid|Targe
ANA|ElMMo3|microrna.org|MirAncesTar|miRDB|MirMAP|Mirza-G|RNAhybrid|TargetRank|Target

|DIANA|ElMMo3|MirAncesTar|mirbase|MirMAP|miRTar2GO|RepTar|RNAhybrid|TargetScan
Mo3|microrna.org|MirAncesTar|miRDB|MirMAP|MirTar|Mirza-G|RepTar|RNAhybrid|TargetRank
pid|DIANA|ElMMo3|MBStar|microrna.org|MirAncesTar|miRDB|MirMAP|RepTar|RNAhybrid
|DIANA|MBStar|MAMI|MirAncesTar|mirbase|miRDB|MirMAP|miRTar2GO|RepTar|RNAhybrid
|DIANA|ElMMo3|MBStar|microrna.org|MirAncesTar|miRDB|MirMAP|Mirza-G|RepTar|RNAhybrid|
|DIANA|ElMMo3|microrna.org|MirAncesTar|mirbase|miRDB|MirMAP|MirSNP|miRTar2GO|Mirza-
Mo3|MBStar|microrna.org|MirAncesTar|miRDB|MirMAP|Mirza-G|RepTar|TargetRank|TargetSpy
Mo3|MBStar|microrna.org|MirAncesTar|miRDB|MirMAP|MirTar|Mirza-G|RNAhybrid|TargetRank
Mo3|MBStar|microrna.org|MirAncesTar|miRDB|MirMAP|MirTar|miRTar2GO|Mirza-G|RNA22|RNA
|Cupid|DIANA|ElMMo3|microrna.org|MirAncesTar|MirMAP|miRTar2GO|Mirza-G|RNAhybrid|Targ
ANA|ElMMo3|microrna.org|MirAncesTar|miRDB|MirMAP|Mirza-G|RNAhybrid|TargetRank|Target
|DIANA|ElMMo3|MBStar|microrna.org|MirAncesTar|miRDB|MirMAP|miRTar2GO|RepTar|RNA22|T
Mo3|MBStar|MirAncesTar|miRDB|MirMAP|MirSNP|MirTar|miRTar2GO|Mirza-G|RNAhybrid|Targe
|DIANA|ElMMo3|microrna.org|MirAncesTar|mirbase|miRDB|MirMAP|miRTar2GO|Mirza-G|RepTa

|CoMeTa|ElMMo3|MBStar|MirAncesTar|miRDB|MirMAP|MirSNP|MultiMiTar|RepTar|RNAhybrid
ANA|ElMMo3|MBStar|microrna.org|MirAncesTar|miRDB|MirMAP|Mirza-G|RNA22|RNAhybrid|Tar
ANA|ElMMo3|microrna.org|MirAncesTar|MirMAP|Mirza-G|RepTar|RNAhybrid|TargetRank
Mo3|microrna.org|MirAncesTar|miRDB|MirMAP|MirSNP|miRTar2GO|RepTar|RNAhybrid|TargetRa
|CoMeTa|DIANA|ElMMo3|MBStar|MirAncesTar|MirMAP|miRTar2GO|Mirza-G|MultiMiTar|RepTar|
|Cupid|DIANA|ElMMo3|microrna.org|MirAncesTar|miRDB|MirMAP|RepTar|RNAhybrid|TargetRan

|CoMeTa|ElMMo3|MAMI|MirAncesTar|mirbase|miRDB|MirMAP|MultiMiTar|RepTar|RNAhybrid
|CoMeTa|DIANA|MirAncesTar|mirbase|MirMAP|miRTar2GO|MultiMiTar|RepTar|RNAhybrid
|DIANA|ElMMo3|MBStar|microrna.org|MirAncesTar|miRDB|MirMAP|MirTar|Mirza-G|RNAhybrid|T

|DIANA|ElMMo3|MirAncesTar|miRDB|MirMAP|miRTar2GO|Mirza-G|RepTar|RNAhybrid|TargetRa
|CoMeTa|DIANA|ElMMo3|MBStar|microrna.org|MirAncesTar|miRDB|MirMAP|Mirza-G|RepTar|RN
Mo3|MBStar|microrna.org|MirAncesTar|miRDB|MirMAP|Mirza-G|RNAhybrid|TargetRank|TargetS
MMo3|microrna.org|MirAncesTar|MirMAP|MirSNP|Mirza-G|RNAhybrid|TargetRank|TargetSpy
ANA|ElMMo3|MBStar|MirAncesTar|MirMAP|miRTar2GO|Mirza-G|MultiMiTar|RepTar|TargetSpy
|CoMeTa|DIANA|ElMMo3|MBStar|MirAncesTar|miRDB|MirMAP|RepTar|RNAhybrid|TargetRank|Ta
Mo3|microrna.org|MirAncesTar|miRDB|MirMAP|Mirza-G|RepTar|RNAhybrid|TargetSpy
ANA|ElMMo3|MirAncesTar|MirMAP|MirSNP|miRTar2GO|RepTar|RNAhybrid|TargetRank
|DIANA|ElMMo3|microrna.org|MirAncesTar|mirbase|miRDB|MirMAP|Mirza-G|RepTar|RNAhybrid
ANA|ElMMo3|MBStar|microrna.org|MirAncesTar|miRDB|MirMAP|Mirza-G|RepTar|TargetRank|Tar

|DIANA|ElMMo3|microrna.org|MirAncesTar|mirbase|miRDB|MirMAP|Mirza-G|RepTar|RNAhybrid
Mo3|microrna.org|MirAncesTar|miRDB|MirMAP|MirTar|miRTar2GO|Mirza-G|TargetRank
ANA|ElMMo3|MBStar|MirAncesTar|miRDB|MirMAP|MirSNP|miRTar2GO|MultiMiTar|RepTar
|DIANA|ElMMo3|microrna.org|MirAncesTar|miRDB|MirMAP|miRTar2GO|Mirza-G|RepTar|RNAhy
|CoMeTa|DIANA|ElMMo3|microrna.org|MirAncesTar|mirbase|miRDB|MirMAP|miRTar2GO|RepTa
Mo3|MBStar|microrna.org|MirAncesTar|miRDB|MirMAP|MirSNP|Mirza-G|RepTar|RNAhybrid|Targ

Mo3|microrna.org|MirAncesTar|miRDB|MirMAP|Mirza-G|MultiMiTar|RepTar|RNAhybrid|TargetRa
Mo3|MBStar|MirAncesTar|MirMAP|Mirza-G|MultiMiTar|RepTar|RNAhybrid|TargetRank|TargetSpy

Mo3|MirAncesTar|miRDB|MirMAP|MirSNP|miRTar2GO|Mirza-G|RepTar|RNAhybrid|TargetRank

Mo3|microrna.org|MirAncesTar|miRDB|MirMAP|miRTar2GO|Mirza-G|RepTar|RNAhybrid|TargetR
Mo3|microrna.org|MirAncesTar|miRDB|MirMAP|MirSNP|MirTar|Mirza-G|RNAhybrid|TargetRank|T
|DIANA|ElMMo3|MBStar|microrna.org|MirAncesTar|mirbase|miRDB|MirMAP|RepTar|RNAhybrid
MMo3|microrna.org|MirAncesTar|miRTar2GO|PACCMIT|RepTar|RNAhybrid|TargetRank
Mo3|MBStar|microrna.org|MirAncesTar|miRDB|MirMAP|MirTar|Mirza-G|RNAhybrid|TargetRank
ANA|ElMMo3|MBStar|microrna.org|MirAncesTar|miRDB|MirMAP|MirTar|Mirza-G|RepTar|RNAhyb
|CoMeTa|DIANA|ElMMo3|MBStar|microrna.org|MirAncesTar|miRDB|MirMAP|MirTar|Mirza-G|RN
|DIANA|ElMMo3|MirAncesTar|miRDB|MirMAP|miRTar2GO|RepTar|RNAhybrid|TargetRank
Mo3|MBStar|MirAncesTar|MirMAP|miRTar2GO|Mirza-G|RepTar|RNAhybrid|TargetRank|TargetSp
|CoMeTa|DIANA|ElMMo3|microrna.org|MirAncesTar|miRDB|MirMAP|Mirza-G|RepTar|TargetRan
|CoMeTa|DIANA|ElMMo3|microrna.org|MirAncesTar|miRDB|MirMAP|MirSNP|MirTar|Mirza-G|RN
Mo3|microrna.org|MirAncesTar|miRDB|MirMAP|miRTar2GO|RepTar|RNAhybrid|TargetRank

Mo3|microrna.org|MirAncesTar|miRDB|MirMAP|miRTar2GO|Mirza-G|RepTar|TargetRank
|DIANA|ElMMo3|MirAncesTar|MirMAP|miRTar2GO|Mirza-G|MultiMiTar|RepTar|RNAhybrid|Targe
|CoMeTa|DIANA|ElMMo3|MBStar|MirAncesTar|miRDB|MirMAP|RNAhybrid|TargetScan
|DIANA|ElMMo3|MBStar|microrna.org|MirAncesTar|mirbase|miRDB|MirMAP|Mirza-G|MultiMiTar
|CoMeTa|DIANA|ElMMo3|microrna.org|MirAncesTar|miRDB|MirMAP|miRTar2GO|Mirza-G|RNAh
Mo3|MBStar|microrna.org|MirAncesTar|miRDB|MirMAP|miRTar2GO|Mirza-G|RNAhybrid|TargetR
|DIANA|ElMMo3|MBStar|microrna.org|MirAncesTar|MirMAP|miRTar2GO|Mirza-G|MultiMiTar|Re

|DIANA|ElMMo3|MirAncesTar|miRDB|MirMAP|MirSNP|miRTar2GO|Mirza-G|RepTar|TargetRank
ANA|ElMMo3|MirAncesTar|MirMAP|miRTar2GO|MultiMiTar|RepTar|RNA22|RNAhybrid
|DIANA|MBStar|microrna.org|MirAncesTar|MirMAP|MultiMiTar|RNAhybrid|TargetScan
|DIANA|ElMMo3|microrna.org|MirAncesTar|mirbase|miRDB|MirMAP|miRTar2GO|Mirza-G|RepTa
Mo3|MBStar|microrna.org|MirAncesTar|miRDB|MirMAP|Mirza-G|MultiMiTar|RNAhybrid|TargetRa
|DIANA|ElMMo3|MBStar|microrna.org|MirAncesTar|miRDB|MirMAP|MirTar|Mirza-G|RepTar|Targ
IA|ElMMo3|MirAncesTar|MirMAP|miRTar2GO|Mirza-G|MultiMiTar|RepTar|RNA22|RNAhybrid
ANA|ElMMo3|MBStar|microrna.org|MirAncesTar|miRDB|MirMAP|Mirza-G|RNAhybrid|TargetSpy
ANA|ElMMo3|microrna.org|MirAncesTar|miRDB|MirMAP|miRTar2GO|Mirza-G|RNAhybrid|Target
|DIANA|ElMMo3|microrna.org|MirAncesTar|miRDB|MirMAP|miRTar2GO|Mirza-G|RepTar|RNA22|
Mo3|MBStar|microrna.org|MirAncesTar|miRDB|MirMAP|MirSNP|MirTar|Mirza-G|TargetRank
Mo3|microrna.org|MirAncesTar|miRDB|MirMAP|Mirza-G|RNAhybrid|TargetRank|TargetSpy
Mo3|MBStar|microrna.org|MirAncesTar|miRDB|MirMAP|Mirza-G|RNAhybrid|TargetScan

|DIANA|ElMMo3|MAMI|microrna.org|MirAncesTar|mirbase|miRDB|MirMAP|Mirza-G|RepTar|RNA
Mo3|microrna.org|MirAncesTar|miRDB|MirMAP|MirTar|miRTar2GO|Mirza-G|RepTar|RNAhybrid
|DIANA|ElMMo3|microrna.org|MirAncesTar|miRDB|MirMAP|Mirza-G|RepTar|TargetScan|TargetS

|CoMeTa|DIANA|ElMMo3|MBStar|MirAncesTar|MirMAP|Mirza-G|RepTar|RNAhybrid|TargetScan
Mo3|microrna.org|MirAncesTar|miRDB|MirMAP|MirTar|miRTar2GO|Mirza-G|RNAhybrid|TargetSp

|DIANA|ElMMo3|microrna.org|MirAncesTar|miRDB|MirMAP|miRTar2GO|PACCMIT|RNA22|RNAhy
|ElMMo3|MAMI|microrna.org|MirAncesTar|mirbase|miRDB|MirMAP|Mirza-G|RNAhybrid
|DIANA|ElMMo3|MBStar|microrna.org|MirAncesTar|MirMAP|Mirza-G|RNAhybrid|TargetRank|Tar
Mo3|MirAncesTar|miRDB|MirMAP|miRTar2GO|MultiMiTar|RepTar|RNAhybrid|TargetRank

Mirza-G|MultiMiTar|PACCMIT|RepTar|RNAhybrid|TargetRank|TargetScan|TargetSpy
SNP|MirTar|Mirza-G|MultiMiTar|PACCMIT|RepTar|RNAhybrid|TargetRank|TargetScan

Tar2GO|Mirza-G|MultiMiTar|PACCMIT|RepTar|RNAhybrid|TargetRank|TargetScan
