# Supplementary material for: Circ0083429 Regulates Osteoarthritis Progression via the Mir-346/SMAD3 Axis
Source: Front Cell Dev Biol. 2021 Jan 15;8:579945. doi: 10.3389/fcell.2020.579945 (PMC7843588; doi:10.3389/fcell.2020.579945)
Supplement: Supplementary file 4 [file Data_Sheet_2.PDF]

Primer sequence

gene	forward primer	reverse primer
MMP3	CCTACAAGGAGGCAGGCAAG	CCCGTCACCTCCAATCCAAG
MMP13	TCGGCCACTCCTTAGGTCTT	AAGTGGCTTTTGCCGGTGTA
ADAMTS4	AACGTCAAGGCTCCTCTTGG	TGACAGGATTGCGGATGCTT
ADAMTS5	CCGGAGCCACTGCTTCTATC	ACCCCCACAGAGGTCAAAGA
ACAN	CGTTTGTAGGTGGTGGCTGTG	CGTTTGTAGGTGGTGGCTGT G
COL2A1	CCAGATGACCTTCCTACGCC	TTCAGGGCAGTGTACGTGAAC
smad3	GCTGAAGCGCACTGACCATA	CCCATCCTGTGGGAATGTCG
ACTIN	AGAGCTACGAGCTGCCTGAC	AGCACTGTGTTGGCGTACAG
Circ0083429	GTGATAGGTGTCGCCTTCGT	TCAGACATCGGGCAAAGGTC

microRNA primer

hsa-miR-4685-5p	ACCAGGGCCAGCAGGGAAT
hsa-miR-6837-5p	ACCAGGGCCAGCAGGGAAT
hsa-miR-4707-5p	atatattGCCCCGGCGCGGGCG GGTTCT
hsa-miR-2467-3p	aAGCAGAGGCAGAGAGGCTC AGG
hsa-miR-6735-5p	GAGGGCAGCGTGGGTGTG
has-miR-4436-3p	GAGGGCAGCGTGGGTGTG
hsa-miR-4632-5p	GAGGGCAGCGTGGGTGTG
hsa-miR-4747-3p	AAGGCCCGGGCTTTCCTCC
hsa-miR-874-5p	ttatCGGCCCCACGCACCAGG GTAA
hsa-miR-1306-5p	CCACCTCCCCTGCAAACGTC C
hsa-miR-4676-5p	cgGAGCCAGTGGTGAGACAG TGA
hsa-miR-5001-3p	cgTTCTGCCTCTGTCCAGGTC CTT
hsa-miR-1915-3p	attataCCCCAGGGCGACGCGG CG
hsa-miR-4479	attataCCCCAGGGCGACGCGG CG
hsa-miR-3194-5p	aGGCCAGCCACCAGGAGG
hsa-miR-6879-5p	CAGGGCAGGGAAGGTGGGA G
hsa-miR-6720-5p	TTCCAGCCCTGGTAGGCGC
hsa-miR-6814-5p	TCCCAAGGGTGAGATGCTGC CA
hsa-miR-4420	TCCCAAGGGTGAGATGCTGC CA
hsa-miR-346	cTGTCTGCCCGCATGCCTG
hsa-miR-8080	cGAAGGACACTGGTGTCAAC GGCT
hsa-miR-6735-5p	CAGGGCAGAGGGCACAGGA AT
hsa-miR-4750-5p	CTCGGGCGGAGGTGGTTGA
hsa-miR-4731-5p	TGCTGGGGGCCACATGAGTG
hsa-miR-2355-5p	ccggcATCCCCAGATACAATGG ACAA
hsa-miR-2276-3p	cTCTGCAAGTGTCAGAGGCG AGG
hsa-miR-7112-5p	ACGGGCAGGGCAGTGCA
hsa-miR-611	GAGGACCCCTCGGGGTCTG A
hsa-miR-1538	atatCGGCCCGGGCTGCTGC
hsa-miR-6858-5p	aGTGAGGAGGGGCTGGCAG
hsa-miR-6749-5p	atTCGGGCCTGGGGTTGGG
hsa-miR-760	aCGGCTCTGGGTCTGTGGG
